# Emerging Emulsifiers: Conceptual Basis for the Identification and Rational Design of Peptides with Surface Activity

**DOI:** 10.3390/ijms22094615

**Published:** 2021-04-28

**Authors:** Fabian Ricardo, Diego Pradilla, Juan C. Cruz, Oscar Alvarez

**Affiliations:** 1 Department of Chemical and Food Engineering, Universidad de los Andes, Bogotá 111711, Colombia; fd.ricardo@uniandes.edu.co (F.R.); d-pradil@uniandes.edu.co (D.P.); 2 Department of Biomedical Engineering, Universidad de los Andes, Bogotá 111711, Colombia; jc.cruz@uniandes.edu.co

**Keywords:** emulsifier peptide, peptide design, emulsion, surfactant, surface tension

## Abstract

Emulsifiers are gradually evolving from synthetic molecules of petrochemical origin to biomolecules mainly due to health and environmental concerns. Peptides represent a type of biomolecules whose molecular structure is composed of a sequence of amino acids that can be easily tailored to have specific properties. However, the lack of knowledge about emulsifier behavior, structure–performance relationships, and the implementation of different design routes have limited the application of these peptides. Some computational and experimental approaches have tried to close this knowledge gap, but restrictions in understanding the fundamental phenomena and the limited property data availability have made the performance prediction for emulsifier peptides an area of intensive research. This study provides the concepts necessary to understand the emulsifying behavior of peptides. Additionally, a straightforward description is given of how the molecular structure and conditions of the system directly impact the peptides’ ability to stabilize emulsion droplets. Moreover, the routes to design and discover novel peptides with interfacial and emulsifying activity are also discussed, along with the strategies to address some of their major pitfalls and challenges. Finally, this contribution reviews methodologies to build and use data sets containing standard properties of emulsifying peptides by looking at successful applications in different fields.

## 1. Introduction

**Figure 1:**
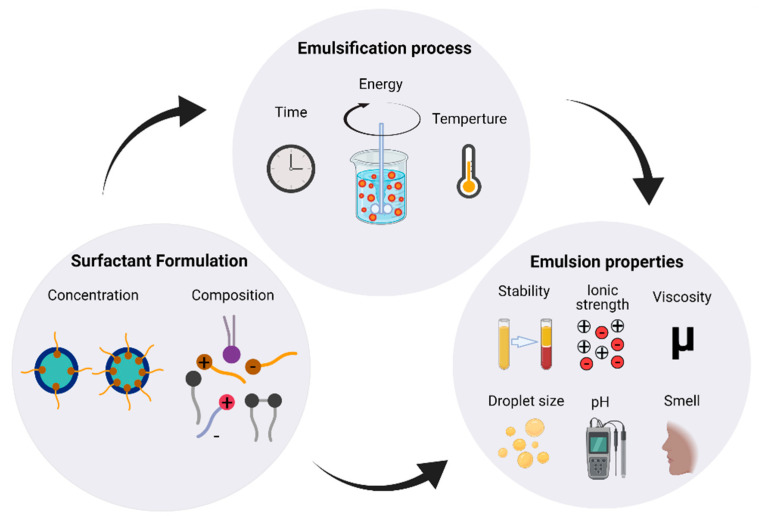
The relationship of the surfactant formulation with the other scales involved in the design of an emulsified product (formulation, process, and properties). The surfactant formulation variables (concentration and composition) are related to the emulsification process variables such as the emulsification time, energy incorporated into the emulsion, and temperature. Additionally, the emulsion properties also depend on the surfactant formulation and the emulsification process variables (created with BioRender^®^, San Francisco, CA, USA).

A microstructure is the type of chemical system in which a consumer product containing an active ingredient is incorporated to achieve an intended functional purpose. Among many others, microstructures (including a wide variety of colloids) can be emulsifiable concentrates, encapsulated granules, capsule suspensions, emulsions, dispersions, wettable powders, and water-dispersible granules [1]. Microstructures are used to formulate products because sole molecules fail to address required attributes according to well-defined customer needs fully and must be mixed with other compounds to produce their principal function, as well as safety, stability, ease of use and applicability, good color and aroma, smoothness, targeted and controlled release, etc. [1,2,3]. For this reason, a wide variety of chemicals are needed for a comprehensive product formulation, but the differences in properties such as state of matter (i.e., solid, liquid, and gas), solubility, and miscibility might cause the coexistence of different phases. Among microstructures, emulsions are one of the most popular for product formulation and perhaps the most relevant for food and cosmetics [3]. Emulsions are also found in paints, textile processing oils, metal cutting oils, pesticides, and pharmaceuticals [4,5]. In emulsions, two immiscible liquids are mixed in a thermodynamically unstable system of droplets dispersed in a continuous phase. The droplets are stabilized with the aid of surface-active species, commonly referred to as emulsifiers or surfactants [4]. Choosing an adequate emulsifier is not a simple task because, besides droplet stabilization, the emulsifier can also affect key process variables such as the necessary energy to be supplied to the system and the total emulsification time [6]. Temperature is another process variable of significant importance when choosing an emulsifier because its overall performance can be modified. Additionally, properties such as viscosity, color, taste, feel, smell, and other variables related to the final product performance are also affected by the surfactant concentration and formulation, as summarized in Figure 1. In this regard, consumers buy a particular product with a list of desired attributes [7]; therefore, the election of the emulsifier is a key factor in the design and final performance of a successful commercial product [3].

Emulsifiers are chemical species that aid in the formation and stabilization of droplets in emulsions, as well as in the stabilization of bubbles in foams and solids in dispersions [8]. Their production may be from petrochemical origin or from biological sources such as bacteria, fungi, or plants [9,10]. There has been an increasing interest to replace emulsifiers of petrochemical origin with bioemulsifiers because of their multiple benefits that include biodegradability, ecofriendliness, and good performance in extreme conditions (e.g., extreme pH conditions, high temperatures, and high salinity environments) [11]. The feedstocks of petroleum are gradually decreasing, and a shortage of this resource in the future is imminent; therefore, the manufacture of products derived from renewable resources is acquiring paramount importance. Furthermore, the volatility of oil prices could, under some circumstances, compensate the high production costs of biosurfactants. In terms of a business model, even when considering the production cost differences and the impact on climate change, premium customers are willing to pay a higher price for biobased products that come from ecofriendly certified industries [12]. For these reasons, bioemulsifier production is an emergent industry that needs the attention and research normally given to other sectors [13].

A major drawback in designing or searching for new bioemulsifiers to be incorporated into improved product formulations is the lack of knowledge about their performance and how to group them into families depending on the required properties and their final application. The majority of studies on emulsifiers have focused on conventional surfactants (i.e., derived from petroleum), and for that reason, understanding the behavior and performance of bioemulsifiers require a much deeper focus, which implies knowledge of the secondary structure at liquid–liquid (oil–water)interfaces, changes in peptide–peptide interactions as a function of the molecular forces, the role of amino acids in the ability to reduce the interfacial tension and stabilize emulsion droplets, and the effect of the system conditions [14]. Moreover, the tools to estimate the performance of conventional emulsifiers, such as the hydrophilic–lipophilic balance (HLB) concept or the group contribution methods to predict the emulsifier properties, are hard to be fully extrapolated to bioemulsifiers [3,14]. One of the main reasons for this is that in conventional emulsifiers the primary structure or the sequence of atoms in the molecule is closely related to its performance; however, in the case of bioemulsifiers (e.g., peptides, proteins), the secondary structural features of the molecule (i.e., α-helix, β-sheet) are the primary factors defining the emulsifying power of the molecule [15].

According to different studies, bioemulsifiers are high molecular weight biomolecules such as polysaccharides, lipopolysaccharides, lipoproteins, and proteins [11,16,17]. However, based on the definition of an emulsifier, short molecules such as peptides and lipopeptides also fall into this category because they aid in the formation and stabilization of emulsions through different mechanisms. In fact, some peptides and lipopeptides exhibit superior interfacial activity than certain conventional commercial emulsifiers [15,18]. For example, the peptide composed by the residues 1–55 from the protein Apomyoglobin shows higher emulsifying activity than its origin protein and the emulsifiers gum Arabic and sodium caseinate. Additionally, the lipopeptide surfactin has higher emulsifying activity than the commercial surfactants sodium dodecyl sulfate (SDS) and Triton X [19,20]. The selection and design of peptides as viable emulsifiers present some advantages over other biomolecules. First, they are smaller in molecular size, and therefore, their performance can be more easily understood and predicted. Second, because peptides are built by blocks of amino acids, they tend to be easily tunable for specific needs by addition, subtraction, or replacement of the residues.

Most recent reviews on bioemulsifiers fail to include peptides as a subcategory of significant relevance [16,17,21]. This review, therefore, provides a comprehensive view of the information needed to understand the performance of peptides with emulsifying properties. Additionally, a thorough discussion is provided on the procedures to discover and design emulsifier peptides. First, the behavior of emulsifying peptides is explained in light of the functional chemical groups present in conventional emulsifiers. Second, the molecular structure–function relationships underlying the emulsifying behavior of peptides are described. Third, the routes used to discover and design emulsifying peptides such as hydrolyzation of proteins, in silico prediction and evaluation tools, de novo design, mimetizing peptides with attractive behavior in similar applications, and manipulating peptides at the O/W interface are addressed. Finally, the challenges and opportunities in the development of novel emulsifying peptides are explained, along with the introduction of a new approach based on the analysis of property databases for the estimation and optimization of the emulsifying behavior.

## 2. Emulsion Formation

**Figure 2:**
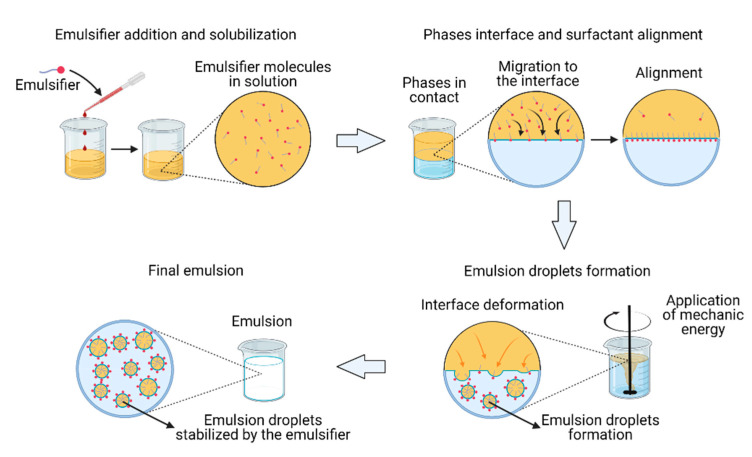
Process of emulsification. During the emulsifier addition and solubilization stage, the emulsifier is added to one of the phases and it dissolves homogeneously in the solution. Next, at the phases interface and surfactant alignment stage, emulsifier molecules migrate to the interface and align their moieties depending on their affinity. The reduction of the interfacial tension allows the interface to deform with the aid of mechanical forces, as shown at the emulsion droplets formation stage. Finally, droplets of one liquid inside the other are formed and stabilized, and therefore, the final emulsion is produced (created with BioRender^®^, San Francisco, CA, USA).

An emulsifier is a substance that aids in the formation and stabilization of droplets to form emulsions. Additionally, a surfactant is a molecule that has the ability to adsorb at an interface/surface and reduce the tension due to its amphipathic structure (presence of hydrophilic and lipophilic domains within its structure) [22]. Surfactants can exhibit emulsifying capabilities when they stabilize emulsion droplets over time by the action of steric, electrostatic, or a combination of both repulsive forces [23]. An emulsifier must have the following characteristics: (i) adsorb preferably at the interface rather than staying in the bulk of the fluid and reduce the tension, (ii) form a condensed layer at the interface that depends on the partitioning ratio, and (iii) reduce the interfacial tension in the same time scale that the emulsion is produced [4]. The action of an emulsifier in emulsion formation can be described as follows. The molecule, which is initially dissolved in one of the phases (i.e., organic or aqueous), diffuses to the interface when they are in contact because the polar and nonpolar portions of the emulsifier show individual affinities for the aqueous and organic phases, respectively. Before the surfactant is adsorbed at the interface, the magnitude of the interfacial tension is proportional to the repulsion between the molecules of the liquids and high values of interfacial tension imply a hard-to-deform interface. When the surfactant partitions at the interface, this repulsion decreases because the surfactant molecules have an affinity for both phases. Consequently, the interfacial tension is reduced, and the interface becomes easily deformed by the action of mechanical forces, thereby forming dispersed droplets of one liquid into the other (i.e., an emulsion) [24,25]. In terms of time scales, the migration and penetration of an emulsifier surfactant into the interface must occur rapidly to avoid coalescence processes that are typically triggered as soon as the droplets start to form [26]. Finally, once the emulsion is produced, the emulsifier molecules must remain at the interface to form a closed-packed layer with high film elasticity where the steric and electrostatic interactions between emulsifiers in neighboring droplets prevent flocculation, Ostwald ripening, and coalescence [26]. The schematic process of emulsion formation is shown in Figure 2.

## 3. Molecular Characteristics of Emulsifying Peptides

During the emulsion manufacturing process, it is important to know the molecular characteristics that make a surfactant molecule suitable as an emulsifier. The prediction of steric and electrostatic forces responsible for emulsification based on the molecular structure is not a simple task, because they depend on multiple factors including the substances present in each phase (that may alter the affinity of the emulsifier), pH, ionic force, temperature, energy incorporated into the system, type of mixing device, etc. Even though the surfactant performance as an emulsifier is highly dependent on the specific system, the reduction of the surface tension is the starting point to assess whether a candidate might have emulsifying potential or not. The surface tension or the tension at the air–water interface is used as an analogy of the interfacial tension between two liquids because air has hydrophobic properties, and the air–water + surfactant system is a more standard model to make comparisons of the behavior among different surfactants. After confirming surface tension reduction, the candidate molecule is evaluated in its ability to stabilize emulsion droplets. This evaluation can be achieved by different experimental methods such as the measurement of the emulsion stability (ES), which consists of turbidity measurements for samples diluted 100 times in a 0.1% SDS-water solution as a function of time, the observation of the coalesced volume, and the evolution of the mean droplet size over time [27,28,29].

The relationship between molecular structure and surface tension reduction of a surfactant remains elusive. The fact that a molecule has polar and nonpolar groups in different moieties does not itself pertain to surface activity. For example, 1-decanol has a terminal hydroxyl group and a hydrophobic tail; however, it is known that this alcohol shows no surface activity. In contrast, 1,2-decanediol, which has a very similar structure but includes one additional hydroxyl group, is able to form micelles and reduce the surface tension of water from 72 mN/m to 23.2 mN/m at the critical micelle concentration (CMC) and 25 °C [4,30]. Normally, in molecules with the presence of amine, carboxyl, and ether groups, together with a hydrophobic portion fail to provide surface activity, and therefore, additional chemical features might be needed to induce surface activity. For example, the combination of one or various ether groups with a hydroxyl group separated by two carbons forms the widely used family of polyethoxylated nonionic surfactants [31]. The presence of an amine and various hydroxyl groups separated by two carbons in the polar section of the molecule constitutes the hydrophilic heads for mono and di ethanolamine moieties present in various surfactants [25]. The amine and carboxylic acid groups separated by two carbons are also combined in surfactants such as *n*-dodecyl-beta-alanine, a molecule that reduces the surface tension of pure water up to 30 mN/m at the CMC and 25 °C [32]. The contrary happens for molecules that contain salt groups, such as carboxylates, sulfonates, sulfates, ammoniums (primary, secondary and tertiary), phosphoniums, and phosphates. The presence of these groups alone confers molecules with surfactant properties as long as they promote hydrophobic interactions with other surfactants and with the molecules present in the organic phase. These interactions are typically achieved by linear or branched nonpolar chains with more than 8 carbons [4].

**Figure 3:**
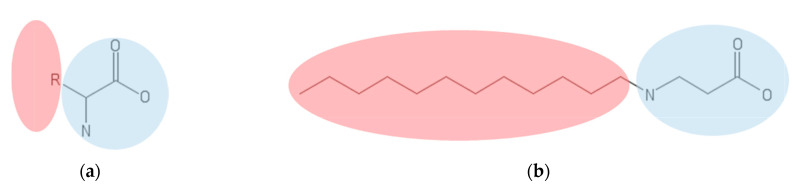
Distribution of the hydrophilic head (blue) and hydrophobic tail (pink) in (**a**) amino acids and (**b**) dodecyl-β-alanine. The hydrophilic head of both molecules have a similar chemical structure, but the amino acids have no surfactant activity while the dodecyl-β-alanine has the ability to reduce the tension of air–water interfaces.

**Figure 4:**
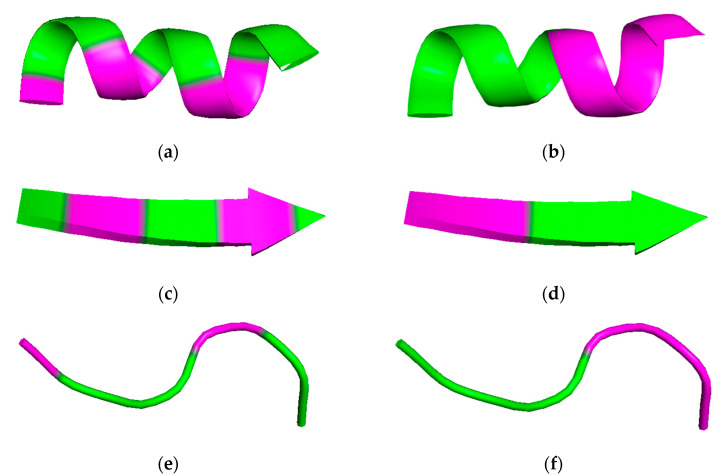
Secondary structures of amphipathic peptides: (**a**) facial α-helix, (**b**) perpendicular α-helix, (**c**) facial β-sheet, (**d**) perpendicular β-sheet, (**e**) facial unordered, and (**f**) perpendicular unordered. Green and purple sections represent hydrophilic and hydrophobic portions, respectively (created with PyMol 2.4.0^®^, New York, NY 10036-4041, USA).

These chemical features might serve as the basis to analyze the structure–performance relationship of amino acids to attempt to evaluate whether a surface activity is present. Based on molecular structure, the surfactant behavior of peptides could, to some extent, be predicted by the presence of the hydrophilic amine and carboxyl head groups. A similar structural configuration has been observed for dodecyl-β-alanine (Figure 3). However, the hydrophobic portion of amino acids is not as large as that of dodecyl-β-alanine, which results in weak hydrophobic interactions, and consequently, the absence of a condensed layer at the interface is generally responsible for providing adequate surface activity properties. Consequently, the incorporation of amino acids in water solutions fails to reduce the surface tension significantly, and therefore, there is a need for very robust methods capable of identifying almost imperceptible changes [33,34,35]. However, despite the amino acids’ limited surface activity, emulsions can be formed with them but with a mechanism that is most likely different from the interfacial tension reduction ascribed to conventional surfactants [36]. Experiments have shown that amino acids apparently diffuse to the interface of emulsions, but the increment of stability over time is negligible [37]. Amino acids fail to provide the molecular-level characteristics necessary to lower the surface or interfacial tension and stabilize emulsions. However, the covalent coupling of several amino acids in chains or peptides can provide emulsifying characteristics because a driving force resulting from the summation of all interactions, namely, hydrophobic, hydrophilic, electrostatic, aromatic (π–π stacking), hydrogen bonds, and van der Waals forces, appears to start playing a significant role [38]. The number of amino acids in a peptide ranges from 2 in dipeptides to about 50 for oligopeptides, a limit above which the amino acid chain is considered to be a protein [39]. Not all peptides have surface properties, because they strongly depend on the difference in hydrophobicity of the amino acids. There are 20 common amino acids occurring in eukaryotic cells whose hydrophobicities have been categorized according to the hydrophobic scale into hydrophobic and hydrophilic [40]. In the case of hydrophilic, amino acids with uncharged and charged polar side chains are the prevalent ones [41]. Similar to how the amphipathicity of conventional surfactants is evaluated by the identification of polar and nonpolar chemical groups in different moieties of the molecule, the amphipathicity of peptides is identified by the location of the polar and nonpolar amino acids in their secondary structural conformation. However, in the case of peptides, this analysis is much more complex because they may exhibit different conformations depending on how hydrophilic and hydrophobic amino acids are intercalated in the sequence and their propensity to adopt certain 3D configurations as well as environmental conditions such as temperature, salinity, pH, and concentration [15,42]. Additionally, the conformations of peptides at interfaces are sometimes different from those in solution, which is why their study requires the application of other experimental techniques to directly probe the interface. Additionally, this can be addressed in silico via molecular dynamics (MD) simulations [15,43,44]. For a peptide to exhibit surfactant capabilities, hydrophilic and hydrophobic moieties are required regardless of the conformation adopted. It is then expected that the nonpolar amino acids will align to the oil phase, while the polar regions will show a preference for the aqueous phase. The types of secondary structural features of amphiphilic peptides are mainly α-helices, β-strands, and unordered; additionally, each one has two possible distributions of the amphipathicity, i.e., facial or perpendicular [18]. The facial distribution of α-helices indicates that the hydrophobic motifs are located in a lateral side of the helix, while the hydrophilic amino acids in a plane opposite to them. This amphipathicity is achieved when hydrophobic residues locate three or four hydrophilic amino acids apart. The facial distribution in β-strands indicates that hydrophilic and hydrophobic residues are placed in the opposite faces of the strand; this can be set by intercalating hydrophilic and hydrophobic amino acids in the peptide. Finally, the perpendicular amphipathicity is produced when the hydrophilic and hydrophobic residues are stacked at the different ends for any conformation [45]. These amphipathic conformations are presented in Figure 4. The ability to reduce the surface tension tends to be greater in short peptides even when one amino acid alone fails to produce such reduction. The size of dipeptides and tripeptides is not large enough to form secondary structures such as α-helixes and β-strands, but they present perpendicular amphiphilicity, which could be the reason why they have surface activity. For example, in the peptide YF, tyrosine (Y) is a hydrophilic residue and phenylalanine (F) hydrophobic; therefore, it presents a perpendicular amphipathicity that is capable of lowering the surface tension in water solution to 48.5 mN/m at the CMC and 25 °C. The peptide VTV has two hydrophobic valines (V) flanking one tyrosine, which allows the peptide to have amphipathicity to lower the surface tension of air–water interfaces at the CMC and 25 °C to 52.3 mN/m [46]. The tendency to be more effective in reducing the surface tension and the relation to size can be explained as follows: first, larger molecules tend to occupy more interfacial area and form loosely packed interfacial layers, which reduces the adsorption effectiveness [4]. Second, large molecules such as bioemulsifiers tend to diffuse slower to the interface and to undergo a lengthy unfolding therein as the hydrophobic domains are exposed to the oil phase, therefore leading to a slower surface tension reduction as well [21]. In general, for biosurfactants, an effective value of the surface tension in water solutions is 35 mN/m, while for conventional surfactants, this value can reach 15 mN/m at 25 °C, excluding the case of surfactants that are used for achieving ultralow surface tension values (10^−1^–10^−5^ mN/m) [9,47]. Values of different parameters related to the interfacial activity of peptides are listed in Table 1.

The formation of a rigid or condensate layer at the interface is needed to stabilize emulsions because strong interfaces are more difficult to coalesce. Additionally, steric and electrostatic interactions hinder the contact between droplets and create electrostatic repulsions that keep them apart [48]. The interaction between peptides has been studied and demonstrated by the tendency to produce supramolecular structures at the interface and in solution, moieties such as fibrils, nanotubes, nanospheres, tapes, and vesicles have been identified as structures that could be the peptide analogs of micelles in conventional surfactants [45,49,50,51,52]. These interactions are very important to stabilize emulsion droplets and can be strengthened by increasing the number of peptide residues. For this, some authors have stated that the minimum number of amino acids in an emulsifier peptide must be 16, while others claim that minimal molecular mass should be around 2000 Da [15,53,54,55]. Longer peptides increase the tendency to stabilize emulsions by forming more rigid interfaces and conformations (i.e., α-helices and β-sheets) that increase the steric repulsive forces with the peptides located in neighboring droplets. It appears that a balance should exist concerning the size of an emulsifier peptide because amphipathic short peptides are more efficient to lower the surface tension, but their ability to stabilize emulsions by peptide–peptide interactions is significantly lower. In general, it is considered that large molecules such as proteins, polysaccharides, lipopolysaccharides are not as effective in reducing the surface and interfacial tension as lower molecular weight surfactants [16,56,57]. In contrast, some works have reported on the ability of short peptides (i.e., dipeptides and tripeptides) to stabilize emulsions; however, they show a different mechanism, because in such case, stabilization proceeds by forming self-assembled nanostructures such as fibers that work as droplet coatings [51,52]. In other words, this mechanism resembles microencapsulation, as opposed to conventional stabilization by steric or electrostatic interactions and therefore it is out of the scope of this review. The type of secondary structural conformation adopted by the peptide at the interface also affects its ability to stabilize emulsions because α-helices and β-strands have shown to provide more stabilization than unordered conformations [58]. The reason is that in more ordered conformations, the peptide–peptide interactions increase, thereby forming a stronger interfacial layer [48]. Additionally, α-helices are suggested to be more useful for emulsification because they have better solubilities in aqueous media than β-sheets [15].

**Table 1:** Properties of interfacial active peptides.

Peptide Sequence	Evaluation Method	Value	References
Ac-MKQLADS LHQLARQ VSRLEHA-CONH_2_	Surface tension at 25 °C, pH 7.4 and a peptide concentration of 5 µM (mN/m)	52	[59]
Ac-MKQLADS LHQLAHK VSHLEHA-CONH_2_		53.1	[59]
Ac-MKQLADS LMQLARQ VSRLESA-CONH_2_		51.5	[59]
Ac-LMQLARQ-MKQLADS-LMQLARQ-VSRLESA-CONH_2_	Interfacial tension (mN/m) in the system octane-water. The concentration of the peptide was 4.5 µM and the interfacial tension without peptide was 51 mN/m.	13.5	[60]
YF	γ_CMC_ (mN/m) at 25 °C	48.5	[46]
VTV		52.3	[46]
FEFRFEFR		52.5	[61]
FEFKFEFK		47	[61]
AEAKAEAKAEAKAEAK		57	[62]
LEELLEELLEELLEEL	Surface tension (mN/m) at pH 7, a peptide concentration of 0.001% (*w*/*v*) and 25 °C	56.8	[15]
	Surface tension (mN/m) at pH 5.5, a peptide concentration of 0.001% (*w*/*v*) and 25 °C	40.51	[15]
ELELELELELELELEL	Surface tension (mN/m) at pH 7, a peptide concentration of 0.001% (*w*/*v*) and 25 °C	54.3	[15]
	Surface tension (mN/m) at pH 5.5, a peptide concentration of 0.001% (*w*/*v*) and 25 °C	49.51	[15]
LELLEEELLEEELLEL	Surface tension (mN/m) at pH 7, a peptide concentration of 0.001% (*w*/*v*) and 25 °C	65.5	[15]
	Surface tension (mN/m) at pH 5.5, a peptide concentration of 0.001% (*w*/*v*) and 25 °C	55.01	[15]
RELEELNVPGEIVESLSSSEESITR	Surface tension at pH 7 (mN/m). The concentration of the peptide was 0.05% (*w*/*v*)	56.1	[55]
	Surface tension at pH 3 (mN/m). The concentration of the peptide was 0.05% (*w*/*v*)	51.8	
YQEPVLGPVRGPFPIIV	Surface tension at pH 7 (mN/m). The concentration of the peptide was 0.05% (*w*/*v*)	54.4	[55]
	Surface tension at pH 3 (mN/m). The concentration of the peptide was 0.05% (*w*/*v*)	50.1	
LSFNPTQLEEQCHI	Presence at the interface of the hexadecane−water system at 20 °C	Present	[54]
YSLAMAASDISLLDAQSAPLRVYVEELKPTPEGDLEILLQKW		Present	[54]
SLAMAASDISLL		Present	[54]
VYVEELKPTPEGDLEIL		Present	[54]
VYVEELKPTPEGDLEILLQK		Present	[54,63]
WENGECAQK	Presence at the oil−protein aqueous solution interface	Present	[63]
IIAEK		Present	[63]
IDALNENK		Present	[63]
VLVLDTDYKK		Present	[63]
ALK		Present	[63]
ALPMHIR		Present	[63]
LIVTQTMK		Present	[63]
GKNHDTGVSPVFA	Interfacial tension (mN/m) for the system: 25% dodecane 75% crude oil (*w*/*v*)/aqueous solution at pH 7 and 1 M of NaCl. The concentration of the peptide was 550 ppm in the oil phase and the clean interface interfacial tension value was 45 mN/m	30	[64]
DPKDGSVVVL		42.7	[64]
TGNTCDNVKQR		32	[64]
THENQLGAGAFG		37.5	[64,65]
QRAALIDCLAPDRRV		39	[64]
QRAALIDCLA		38	[64]
ILEFLEGQLQEVDN	Interfacial tension (mN/m) with Medium Chain Triglycerides (MCT) oil. A peptide concentration 0.2%wt. was used in the aqueous phase with an adjusted pH of 7 with a buffer solution. The interfacial tension of the clean interface was 26 mN/m	22.41	[18]
KYDGKYLMQVLQE		18.86	[18]
KYLMQVLQEKL		17.87	[18]
KKPVSKDSPETYEEALKRFAKLLSDRKKL		16.93	[18]
EALKRFAKLLSD		16.83	[18]
AKDIVPFYFEHGPHIFN		16.63	[18]
KYLMQVLQEKLGE		16.48	[18]
IPATILEFLEGQLQEVDNN		15.4	[18]
DSPETYEEALKRFAKLLSD		14.28	[18]
NRPFAAAKDIVPFYFEHGPHIFN		14.13	[18]
DDNFCAKVGVVIQ		24.72	[18]
LNIQFNI		22.04	[18]
GKELDPRLSYRI		21.12	[18]
LGGDVYLGKSPNSDAPCP		19.33	[18]
VHQNGKRRLALVKDNPLDVSFK		18.11	[18]
FIPLSTNIFEDQLLNIQFNIPT		14.89	[18]
CPFSSDDQFCLKVGV		13.97	[18]
IGSSSHFGPHIFEGELLNIQFDIS		13.62	[18]
ELDSRLSYRIISTFWGALGGDVYLGKSPN		12.12	[18]
ELDSRLSYRIISTFWGALGGDVYL		12.09	[18]
LNIQFNIPTPKLC		12.07	[18]
TPNENNRPFAAAKDIV		23.11	[18]
FAKLLSDRKKLRANK		21.81	[18]
VGVVIQNGKRR		21.34	[18]
NPNSSYRIISI		20.78	[18]
CRDDNFCAKVGVVI		20.14	[18]
LLTAMITTPNENNRP		19.67	[18]
RDDNFCAKVGVVI		18.15	[18]
DNFCAKVGVVIQNGKRR		18.16	[18]
FCLKVGVIHQNGKRRLALVK		17.64	[18]
DTNGKELNPNSSYRIISIGRGALGGDVYL		17.41	[18]
FCLKVGVVHQNGKRRLALVKDNP		17.26	[18]
HQNGKRRLALV		15.26	[18]
SSDDQFCLKVGVV		15.07	[18]
FDVIGGTSTGGLLTAMITTPNENNRP		13.73	[18]
KDNPETYEEALKRFAKLLS		13.74	[18]
GIIPATILEFLEGQLQEVDNN		13.22	[18]
GIKGIIPAIILEFLEGQLQEVDNNKDAR		10.84	[18]
IRPIPFIPRGGKT-NH_2_	γ_CMC_ (mN/m) at 25 °C	72.01	[66]
FIGALLRPALKLLA-NH_2_		46.9	[66]
GLKEVAHSAKKFAKGFISGLTGS		72.01	[66]
LKGASKLIPHLLPSRQQ		72.01	[66]
FIGALLGPLLNLLK-NH_2_		38.3	[66]
IRPVPFFPPVHAKKVFPLH		72.01	[66]

## 4. Impact of Emulsion Conditions on the Peptide Emulsifying Behavior

In addition to the structure–performance relationships provided above for understanding the behavior of emulsifying peptides, their performance also depends on the conditions of the system. The functionality of a peptide is strongly influenced by the secondary structure as a result of intermolecular forces. These forces are largely dependent on the emulsion conditions; therefore, as these conditions change, the interactions that dictate the affinity of the peptide for the involved phases and peptide–peptide interactions might be significantly altered. Consequently, the behavior of a peptide emulsifier is affected by the pH, electrolyte concentration, and temperature [67]. The effect of the pH is similar to that of conventional emulsifiers since it depends on the isoelectric point (the value of pH in which the net surface charge of the molecule is zero), which in turn, is altered by the presence of individual amino acids and by their interactions [68]. It is reported that peptides near the isoelectric point, which is normally located in the acid range of pH, have limited emulsifying properties [69]. At the molecular level, the change of pH alters the charge of chemical ionizable groups present in amino acids such as amino, guanidino, or imidazole. Such charge will exert repulsion over other atoms in the sequence, but the net sum of all interactions must provide a suitable charge balance in which emulsion droplets stay apart while avoiding a decrease in the interfacial film strength, which is largely responsible for stability. Additionally, the change of repulsive forces in the peptide may destabilize the conformation, also affecting the stability of the emulsions. The interplay of interactions is complex because of the different number and type ionizable groups that might be present in a particular sequence [48]. Since at the isoelectric point, the net charge of the peptide equals zero, there is no repulsion between surfactant molecules located in adjacent droplets, thereby promoting coalescence [70]. This mechanistic description explains why some peptides form stable emulsions in a certain pH range while others work better in a completely different one [15,69,71].

Concerning the ionic strength, the results are diverse. In some studies, emulsions made with proteins have shown a marked tendency for destabilization by the addition of electrolytes such as NaCl, KCl, or CaCl_2_. The justification for this behavior is that ions produce a screening effect of the electrostatic repulsive interactions between peptides located in different droplets, therefore, repulsion is reduced, and the droplets are more prone to coalescence [72,73]. This analysis can be also extrapolated to peptides since they also have functional groups that might cause repulsion with other peptide molecules, and they can also be screened by electrolytes. In this regard, experiments with peptides show that the presence of NaCl and CaCl_2_ reduces the ability of peptides to stabilize emulsions [74]. Although electrolytes can disrupt ionic interactions between peptides, interactions responsible for stabilizing secondary structural features are also modified, thereby leading to different conformations. New conformations can increase or reduce the stability depending on the newly developed distribution of interaction forces. For example, experiments using peptide fractions obtained from whey protein have demonstrated that an increase in the ionic strength of the emulsions improved the stability, a fact that was attributed to the exposure of hydrophobic domains to the oil phase, which, in turn, led to an increase in the affinity of the peptide for the interface and in the strength of this interface [69]. In conclusion, more insight is needed into the effect of ionic strength on the stability of emulsions, a correlation that is presumably dependent on the chemical groups of the peptide and its conformation at the interface. Finally, the temperature may detrimentally impact the stability of emulsions because it induces secondary structural changes in the peptides. In this regard, temperatures above 40 °C disrupt the stability of α-helices and β-sheets [48].

## 5. Methodologies for Screening and Designing Emulsifying Peptides

Some experimental and in silico strategies have been used to identify peptides with emulsifying capabilities. In other words, peptides with the ability to lower the interfacial tension and stabilize emulsion droplets. Additionally, other studies have focused on selecting or designing the best emulsifier peptides for a certain application. Both types of studies will be discussed below.

### 5.1. Identification of Emulsifying Peptides from Hydrolyzed Extracts of Proteins

Peptides with emulsifying behavior have been discovered, produced, and isolated from proteins. The fragmentation of proteins has been applied to find peptides with different functionalities and bioactivities such as the protection of the intestine gut mucosa [75,76], antiobesity [77], anti-inflammatory [78], antihypertensive [79,80], antimicrobial [81], antiviral [82], foaming [83] and emulsifying power [84]. Since some proteins have been reported to show emulsifying properties, attempts to study their conformations, domains, functionalities, and ways to engineer them for specific applications have led researchers to discover new peptides with emulsifying activity. The process of protein break out into peptides is known as hydrolyzation. Depending on the experimental method, three types of hydrolyzation processes can be identified: chemical, thermal, and enzymatic. Chemical hydrolyzation involves the reaction of the protein with an acid or a base at high temperatures. This process presents different drawbacks such as the lack of reproducibility, low specificity, and denaturation of the amino acids [85]. Thermal hydrolysis involves increasing the temperature of protein solutions to break the amide bonds and generate peptides [86]. Additionally, in enzymatic hydrolysis, the protein is cut into peptides by a reaction with a protease, which breaks the peptide bonds out at specific amino acid sites depending on the type of protease, concentration, temperature, and pH of the reaction medium. Additionally, the deactivation method for the protease after the process is completed plays an important role in the final state of the obtained peptides [85,87]. Proteases are widely available in nature, but some frequently used include chymosin, trypsin, plasmin, alcalase, bromelain and globin [88].

Protein hydrolysis usually provides peptides whose emulsifying activity is even better than that of the original protein, a feature attributed to differences in the structure at the interface such as the increase in the exposure of hydrophobic amino acids, which can potentially have greater interaction with the interface [54,89,90,91,92]. When proteins are hydrolyzed by enzymes, the molecular weight of the formed peptides decreases as the degree of hydrolysis increases. However, it is important to control this degree because the recommended number of residues for emulsifier peptides should be above 20 [53].

**Figure 5:**
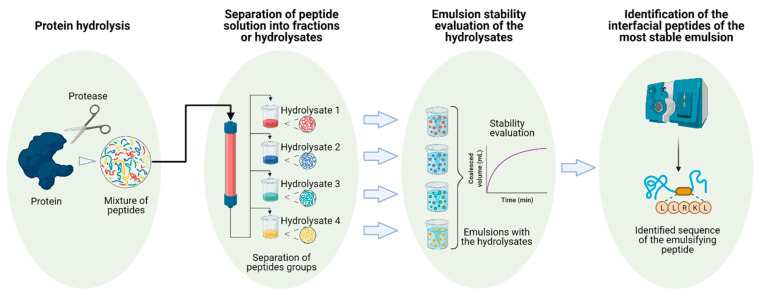
Schematic process for the discovery of emulsifying peptides from proteins. The protein is cut into peptides by a protease. Then, the mixture of peptides is separated by chromatography and different fractions are generated according to their hydrophobicities. Each fraction is then used to produce emulsions whose stability is evaluated. Finally, the most representative peptide contained in the emulsion with the highest stability is identified by proteomics methods (created with BioRender^®^, San Francisco, CA, USA).

The process of finding emulsifier peptides from enzymatic hydrolysis of proteins follows these steps: (i) the protein is divided out by an enzyme, and the resultant solution is separated into fractions or hydrolysates with different hydrophobicities or molar weights using physical methods such as chromatography; (ii) the hydrolysates are tested in their ability to form emulsions; therefore, the fractions with the best performance are selected, and finally; (iii) the chosen fractions are molecularly characterized (generally via mass spectrometry) for the identification of the most abundant peptides contained in each of the collected fractions. A schematic representation of all the steps is shown in Figure 5. A large body of literature has produced hydrolysates of protein with good emulsifying properties, but the majority omitted the stage of peptide identification (step iii), narrowing down the analysis to only their molecular weights or hydrophobicities [83,90,93,94,95,96,97,98,99,100,101,102,103,104,105,106]. Some of the most comprehensive studies involving the three steps mentioned before are discussed next.

Bovine serum albumin (BSA) is a protein whose emulsifying behavior has already been studied and clearly demonstrated in a number of systems including emulsions of *n*-tetradecane–phosphate buffer (pH 7.4), soybean oil–phosphate buffer (pH 7.0), and olive oil–BSA solution (pH 5.5) [107,108,109]. BSA’s residues 377–582 can be produced by hydrolysis with trypsin and have exhibited a marked tendency to adsorb at the oil–water interface; however, the emulsifying activity index (EAI) of the peptide was negligible and a mixture with other small peptides was necessary to improve this property. Additionally, this index was below that of pure BSA, and no other properties related to emulsion stability were measured [110]. Another example of proteins to produce emulsifier peptides is the caseins. These can be found in bovine milk, which has been reported to exhibit interfacial activity, as evidenced by their ability to produce O/W emulsions [53,56,111,112,113]. For this reason, much effort has been invested toward identifying emulsifier peptides from hydrolysates of β-casein. For instance, Girardet et al. [53] used trypsin to hydrolyze the β-casein directly from the emulsion and found that the fraction of the hydrolysate adsorbed at the interface that lowered the surface tension the most was composed mainly by the peptide corresponding to residues 114–169. The same protein was hydrolyzed by chymosin to obtain hydrolysates rich in peptides that corresponded to residues 1–25 and 193–209, which predominantly contained hydrophilic and hydrophobic domains, respectively [55,114]. These peptides exhibited low emulsifying activity at neutral pH values (3–10) but a noticeable activity under acidic or alkaline conditions [55]. Finally, β-Lg is an amphipathic protein present in whey, and its adsorption at oil–water interfaces has been studied for the manufacture of emulsions, especially for food and pharmaceutical applications [89,115,116,117]. Hydrolysis of β-Lg has been carried out using chemical and enzymatic cleavage to obtain several peptides with a tendency to adsorb at hydrophobic interfaces. The identified interfacially active peptides are listed in Table 1. Emulsions were then produced with hydrolysates containing the identified peptides, but the parameters generally measured to describe their ability to form or stabilize emulsions were not reported.

### 5.2. Design of Emulsifying Peptides from Proteins through Computational Tools

From the protein hydrolysis process described before, the knowledge of peptide behavior has increased significantly, and their performance has been described through studies on structure–performance relationships. Performance data of peptides for various functionalities and bioactivities are important to know which peptides are better than others for a particular application and to build relations between this performance and the intrinsic physicochemical properties of the peptide such as size, amino acid composition, sequence, and secondary structure. For this reason, some researchers have searched for common patterns linking amino acid sequences with certain functionalities of interest. For instance, the presence of proline (P) and valine (V) has been linked with antihypertensive properties in peptides [118]. The antimicrobial peptides (AMPs) are usually short (10–15 residues), and they are rich in hydrophobic and cationic residues [119]. The spontaneous membrane translocative peptides (SMTP) are able to penetrate cell membranes without forming pores, which has been attributed to the amino acid sequence “LRLLR” in the positions 5–9 of a 12-residue peptide [120]. Specifically for emulsifying peptides, the α-helix and β-sheet secondary structures along with their amphipathic properties are necessary to have interfacial activity and consequently good emulsifying behavior [18].

Peptide performance data, in combination with the knowledge of relevant structure-function relationships, have allowed the implementation of computational tools and mathematical models in the identification of bioactive and functional peptides from proteins, including emulsifying peptides. Several databases with performance data of many peptides have been built and are continuously revisited and augmented by research groups worldwide. Such computational tools use the databases to develop machine learning predicting algorithms based on the data tendency, structure–function relationships to identify potential peptide candidates with emulsifier activity, and molecular mechanistic functions to simulate the behavior of the molecule within the system. These in silico prediction tools are usually used to support the discovery pipeline of emulsifying peptides from protein hydrolysates (i.e., cleavage, hydrolysate functionality testing, and functional peptides identification). However, in this case, the process steps are rearranged as follows: (i) cleavage and (ii) functional peptides identification or characterization. These in silico methods have shown many advantages, in comparison to experimental approaches, and particularly a significant reduction in time and cost to run the experiments required for protein cleavage, peptide identification, and performance evaluation. This acceleration in analysis has been enabled by the rise of computational power observed over the past two decades, which allows running complex algorithms in reasonable time frames [121]. As a result, it is possible to evaluate many different conditions, which might require much higher (by several orders of magnitude) investments in resources if conducted experimentally. This route, therefore, offers savings in reagents, equipment, intensive labor, services, risks and reduces the possibility of human error while conducting experiments.

For the cleavage step, it is possible to implement algorithms that rely on information about enzyme specificities (the specific bonds cut by the enzyme) and recognition sites (amino acid sequence necessary for enzyme recognition and subsequent cutting) [122]. In consequence, by having the information of the amino acid sequence of a protein or just its name, it is possible to cleave it in silico with a selected enzyme or a chemical compound. The obtained peptides are shown in different formats with the aid of the BIOPEP or PeptideCutter software packages [123,124]. After the protein is cleaved and the peptides generated, the functionality or bioactivity of the peptides can be also evaluated in silico for the desired application. In this regard, several prediction software packages are available to estimate peptides properties such as structure, isoelectric point, molecular weight, grand average hydropathicity, instability index, net charge, aggregation, solubility, and hydrophobicity as well as bioactivities that include toxicity, potential allergenicity, cell-penetrating potential, and protein–peptide binding interactions [118,125,126]. These packages are mainly based on computational methods that quantitatively model the relationship between structure and activity (known as QSAR) by relying on the performance-structure peptides databases [125]. Model developments are generally conducted in three steps: the test training selection, feature selection, and modeling and validation [127]. The test training selection consists of randomly dividing the data set into one group that is used to train the model and another smaller group used to apply the model and validate it. Then, the properties of the substances are selected based on known descriptors that can be structural, sequence based, and physicochemical. The feature selection step consists of reducing the features or making a combination of features so that overfitting of them is prevented. Modeling is the process by which the algorithm learns relationships and tendencies from the database and produces a mathematical model capable to predict property values. The models can be linear such as the multiple linear regression (MLR), ordinary least squares (OLS), principal component regression (PCR), and partial least squares (PLS). Alternatively, they may involve nonlinear approaches such as artificial neural networks (ANNs) and support vector machines (SVMs) [127]. Finally, the model is validated by different methods and metrics. Validation methods can be divided into two groups: internal validation that checks upon the power of the model to fit data used in the model development and external validation, which evaluates the model capacity to predict activities of new molecules. Concerning internal validation methods, the calculation of squared correlation coefficient (R^2^) accounts for the difference between predicted and experimental values. One method is the cross validation, which applies regressions to several data subsets that have one or several molecules excluded (i.e., leave one out or leave many out), then the models are checked by the evaluation of the missing molecules to calculate the cross-validated correlation coefficient R^2^ (Q^2^). Another internal validation method is bootstrapping, which involves the random generation of data subsets in which models are generated and used to evaluate data excluded from the groups. In this case, the parameter Q^2^ is used to account for the precision of the model. Finally, the randomization test scrambles the activities and randomly assigns them to the molecules; if the random predictions of the models are similar to that of the original model, the data are not enough to support the model. Regarding external validation, the most important metric used to evaluate the predicting capacity of the model is the squared correlation coefficient (R^2^_pred_). Additional information on these coefficients calculation and others not commonly used can be consulted elsewhere [128]. The accuracy of these models (R^2^) ranges from 0.5 to 0.998, with the nonlinear models being the most accurate. The prediction success of each model depends on the amount, quality, and diversity of the data, as much as on the number of residues of each peptide in the database [127]. Another method to estimate the properties and behavior of a peptide in a controlled environment is through MD simulations. This approach has gained significant traction as a tool to predict specific properties and secondary structure of peptides under varying conditions of concentration, presence of chemical compounds, and changes in temperature and ionic strength. MD simulations consist of calculations of properties and parameters of molecules by looking at their atomic interactions. In this regard, the Newton’s equation of motion is solved to calculate the spatial trajectories of molecules as they are subjected to an external potential energy or force field for each atom. MD simulations are therefore useful to predict changes in 3D conformations, the interaction of peptides with themselves or with other substances (e.g., water, oil–water interfaces, and biological membranes), and changes in properties with conditions as a function of time [129,130,131,132]. The imposed force fields consist of a set of equations whose parameters can be estimated experimentally or approximated by quantum mechanical calculations. Additionally, the success in the application of MD depends on the used time and length scales, and the agreement of the results with the experimental data [129]. In this regard, good agreement has been reported for peptides in the penetration of lipids bilayer membranes [133,134,135], while significant discrepancies have been observed in the study of water–dodecane interfaces [65]. However, an important body of literature concurred that simulations and experiments should not compete but be complementary, and none of the two is enough for a complete understanding of the phenomena [44,136].

Particularly referring to the emulsification processes, MD simulations have been used for the analysis of many phenomena, including the behavior of polyoxyethylene alkyl ether surfactants in surfactant–water mixtures [137], the change in the emulsifying ability of a surfactant by the supply of CO_2_ or N_2_ [138], the structural features and interactions among cellulose molecules to stabilize octane–water interfaces [139], the effect of temperature on the interfacial properties of surfactant micelles [131], and how the HLB is related to the configuration distribution function of emulsifying peptides [130].

Even though the discovery of emulsifying peptides from proteins can be fully addressed in silico (i.e., the protein cleavage and property evaluation steps), a hybrid/integrated approach is more common, in which some steps are conducted in silico, while others experimentally [122]. One example of such an approach was the discovery of the surfactant potential of the outer membrane protein A (OmpA) of *Escherichia coli* (*E. coli*), which was originally predicted by MD simulations and further tested experimentally in the preparation of dodecane–water emulsions [140]. Additional studies were then dedicated to the identification of emulsifying peptides from OmpA’s hydrolysates, which were then analyzed based on the hydropathic plot of OmpA that helped to predict amphipathic moieties. This analysis led to the identification of 16 possible emulsifying peptides, which were evaluated by MD to find the two peptides whose Gibbs energy was the minimum. The study concluded that indeed these two peptides formed dodecane–water emulsions as predicted by MD. However, MD also indicated that one of them will have better emulsifying behavior, but experimental results showed the contrary. This contradiction was attributed to the time and length scales used for the simulations, which failed to consider the self-assembly of supramolecular structures [65]. For this study, the protein cleaving step was omitted because the protein was only used to identify the emulsifying peptides, and then these were produced by solid-phase peptide synthesis (SPPS). Starchy vegetables such as potatoes contain proteins with nutritional and functional value, which can be used to obtain emulsifying peptides [141]. Particularly, the potato proteins have proved to have emulsifying properties [142]. For this reason, Garcia-Moreno et al. [18] cleaved potato proteins in silico and produced peptides of 7–30 amino acids that were evaluated by an algorithm that took into consideration their amphiphilic nature and potential to form secondary structures, factors that have been related to superior emulsifying behavior. The potential emulsifying peptides identified were then synthesized, and the emulsifying activity and the interfacial tension were evaluated. This study involved the in silico steps for cleaving and functional peptides identification, combined with the experimental step of functional testing, and it was concluded that the emulsifying activity was not “fully” predicted.

### 5.3. Design De Novo of Emulsifying Peptides

Due to the several structure–performance relationships constructed by the identification and testing of emulsifying peptides derived from proteins, several patterns have been discovered in terms of the amphiphilicity, amino acids sequence, and peptides’ length to assure a good emulsifying behavior. Some authors have used some of these relationships to design new emulsifying peptides. Saito et al. [15] noted that most of the identified emulsifying peptides from proteins had α-helical structures and the mandatory presence of hydrophilic and hydrophobic amino acids. Consequently, they chose the amino acids leucine (L) and glutamine (E) to create peptide sequences in which the amino acids were permutated to obtain different amphiphilic structures. The peptides H (LEELLEELLEELLEEL) and S (ELELELELELELELEL) formed mainly α-helixes and β-sheets, while the peptide R (LELLEEELLEEELLEL) showed no amphiphilicity and, consequently, poor emulsifying activity. In this study, it was possible to elucidate that the secondary structure is important at the moment of stabilizing emulsions and, even when the secondary structures were not measured directly at the interface but in solution, only the peptide with no amphiphilicity was not a good emulsifier [15]. Applying the fact that the amphiphilicity improves the emulsifying behavior of peptides, some authors produced emulsions using copolypeptides in which one end has hydrophobic amino acids and the other hydrophilic ones. The peptides tested had the general structure poly(L-lysine*HBr)x-b-poly(racemic-leucine)y, or K_x_(rac-L)_y_, where x = 20, 40, 60, and 100; y = 5, 10, 20, 30; K is the cationic hydrophilic amino acid lysine, and L the hydrophobic amino acid leucine. The results showed that the longer the hydrophobic segment the more stable the emulsion was and the limit for this was the low solubility in water of the longer peptides. The amino acid leucine promotes α-helical structures that interact strongly with themselves making the peptide molecules poorly soluble in water. This is the reason why racemic leucines that have a disordered chain conformation have been used to improve the solubility of the peptides in water. These copolypeptides formed double (W/O/W) emulsions aided by high-pressure homogenizer and had long-term stabilities that approached 1 year when only a short volume fraction creamed. The no racemic peptide K_60_L_20_ failed to form double but instead a stable O/W emulsion [143].

### 5.4. Identification of Emulsifying Peptides by Mimetization

Thus far, the identification of emulsifying peptides can take several routes. Emulsifying peptides are a class of surfactant peptides capable of stabilizing emulsion droplets. However, these peptides have been mostly employed in other applications, including the synthesis of hydrogel scaffolds for cell culture, templates for biomimetic mineralization and nanofabrication, drug delivery, hemostasis, membrane protein stabilization, and antimicrobial agents [41]. There has not been extensive work on emulsifying peptides yet since it has for other classes of surfactant peptides such as the AMPs. These peptides and the emulsifying ones have an important characteristic in common; in both, it is necessary that its hydrophobic portion strongly interacts with the oil phase, which, in the case of the AMPs, corresponds to the phospholipid rich bacterial membranes [144]. This similarity constitutes an important opportunity to discover emulsifying peptides by analogy with AMPs, which are more extensively studied and thoroughly characterized. An attempt to achieve this was by producing emulsions with the antimicrobial peptide A_9_R as the stabilization happened by assembling supramolecular structures over the droplets [145]. Even when stabilization most likely proceeded by an atypical nonemulsifier-like mechanism such as the formation of supramolecular structures, larger AMPs with different amphipathicity distributions have also shown abilities to reduce the surface tension. A more comprehensive performance evaluation as emulsifiers might yield useful results [146].

### 5.5. Design of Emulsifying Peptides by the Crosslinking of Peptides at the Interface

Another novel route to design emulsifying peptides was proposed by Dexter et al. [45]. Their approach starts with the identification of peptides with facial amphipathicity and the ability to reduce the interfacial tension by insertion into the interface of the two immiscible liquids. This reduction eases the formation of the emulsion with no guarantees over stability. To address this issue, the mechanical strength of the interfacial layer was increased by stronger interactions between peptide molecules through changes in pH, oxidation/reduction, and metal ion chelation [48]. Examples of this approach are discussed below.

Lac 21 (Ac-MKQLADS LMQLARQ VSRLESA-CONH_2_) is a peptide that corresponds to the residues 339–359 of the Lac repressor protein present in bacteria in which the first three amino acids PRA were replaced by MKQ. This peptide’s capacity to form tetrameters has attracted the attention of researchers since they can gain insights into the mechanistic understanding of folding and stability of proteins [147]. Lac 21 is also highly surface active, as evidenced by a reduction of the interfacial tension of the octane–water interface from 50.1 mN/m to 14.5 mN/m, which is larger than that of proteins such as β-casein, lactoglobulin, and lysozyme [60,148]. Despite this, Lac 21 forms weak films at the interface most likely due to low intermolecular interactions; therefore, the emulsions produced with this peptide are expected to be unstable [45,149]. At the interface, Lac 21 presents an α-helical conformation with facial amphipathicity, which maximizes the contact of the peptide’s hydrophilic groups with the interface. To improve the peptide–peptide interactions, the peptide AM1 (Ac-MKQLADS LHQLARQ VSRLEHA-CONH_2_) was designed by changing the amino acids in Lac 21 at positions 9 and 20 by histidine residues, which have the capacity to bind to metals. As a result, by exposure to metal cations (e.g., Zn(II)) the peptides’ helixes crosslink, and consequently, the interface becomes stronger, in other words, it turns a detergent state into a cohesive film state [150]. When this state is achieved, the stability of emulsions is significantly prolonged. The coalesced volume observed after a few seconds for the system toluene–water–AM1 can be only noticed for the system toluene–water–AM1+Zn(II) in a lapse of 20 h. Another advantage of this system is that the stability can be reverted by adding a chelating agent or an acid to the stable emulsion. This causes the complex AM1+Zn(II) to destabilize by breaking the metal–histidine bonds, thereby making the interface return to the detergent state [28,150].

The same design method was used for the peptide AFD4 (Ac-MKQLADS LHQLAHK VSHLEHA-CONH_2_), which was obtained by the replacement of amino acids in positions 9, 13, 17, 20 of Lac21 with histidine residues. In this case, four residues can be crosslinked with transition metal ions such as Zn(II), Ni(II), and Co(II) to enable stronger films at the interface. Dodecane emulsions developed with AFD4 and Zn(II) showed good visual stability for up to 30 days. The interface formed by AFD4 was even stronger than that of AM1, but when compressing a droplet, the interface showed a marked tendency to form wrinkles. This behavior has been typically observed for solid coatings, as opposed to the highly mobile interfaces formed by small molecular size surfactants. The high stability of the emulsion and the solid-like appearance of the interface can be easily reverted as described for AM1. In this case, spherical droplets of low stability are formed again without significantly altering the interfacial tension [59,151]. The interfacial tension values achieved for crosslinked peptides via chelating agents are shown in Table 1.

## 6. Challenges and Opportunities

The selection of an emulsifier peptide for a certain application has several limitations in comparison to the selection of conventional emulsifiers. In this regard, the evaluation of its interfacial activity is generally sufficient for predicting emulsion forming behavior, which can be relatively easily conducted by measuring the interfacial tension. Each one of these methods yields results that are dependent on the conditions of the system, including the salinity and the pH of the aqueous phase, the temperature of the system, the concentration of the emulsifier, and in the case of the interfacial tension, the type of organic phase used. Despite this variety of conditions, the most standard value reported in the literature is the surface tension of the surfactant at the liquid (water)–air surface (i.e., in the absence of electrolytes, acids, or any other substances) at the (γ_CMC_) at 25 °C. This parameter has been reported for many surfactants and is widely used for comparisons. Second, the ability of the surfactant to form emulsions needs to be evaluated. Even though experimental evaluation is necessary because the emulsifier performance is dependent on the ingredients of the emulsion, criteria such as HLB or the hydrophilic–lipophilic difference (HLD) allow predicting the type of emulsion that the emulsifier is likely to form, and in combination with the HLB required by some oils, it is also possible to approximate the structure that the surfactant must possess to provide adequate stabilization. This concept has become extremely useful, and even some surfactant manufacturers usually provide the HLB value along with the surface tension in water solution for each emulsifier in their portfolio.

Concerning emulsifier peptides, an analogous standard parameter for the quantification of the interfacial activity is yet to be defined. First, there are a few studies in which the interfacial tension or the surface tension is measured for one peptide alone. Second, these studies are not just scarce, but they also report on different interfacial properties (e.g., interfacial tension and surface tension) under different conditions, which makes performance comparisons extremely difficult. Typically, the reported parameters have been the surface tension of the peptide in water solution at the CMC as in conventional surfactants [46], the surface tension in water at a given peptide concentration [28,59], and the interfacial tension at different peptide concentrations and pH values [18,60,152]. Moreover, stabilization of emulsion droplets has been evaluated mainly by the measurement of the time that the emulsion requires to visually exhibit phase separation [28,145], and the evolution of the average droplet size as a function of time [18]. None of these methods provide a route for the comparison of different emulsifying peptides because both the process of making the emulsion and the measurement methods are not standardized. This is also the case for the type of oil phase changes and the substances dissolved in the aqueous phase to change the pH and salinity. Additionally, sometimes the temperature is not even reported, and the percentage of dispersed phase often changes. The methods to characterize the stability of the emulsion such as the detection of coalesced volume or phase separation as a function of time rely on visual observations, which are subject to human error and therefore high variability. Furthermore, coalescence is not the only instability mechanism of importance and therefore most studies disregard the information provided by other parameters such as the aggregation of droplets (flocculation) and the accumulation of droplets above or below the emulsion (creaming). The measurement of average droplet size is conducted by taking samples from the emulsion and then read by turbidity instruments. To collect useful data, the system has to be perturbed, which affects the distribution of the droplets, which also depends on the emulsion height as a consequence of the destabilization phenomena and the action of the gravity. As a result, this method provides imprecise information due to the heterogeneity of the samples as they are collected and transferred into the instrument.

Setting standard reliable methods for characterizing emulsifiers can provide important information on properties that are useful to understand structure–performance relationships, a knowledge that can be used in the design and selection of peptides. As an example, in AMPs, the MIC is a standard measurement to evaluate the antimicrobial activity of peptides. The procedure to measure the MIC has even more variables to control and is more intricate than the process of making emulsions and measuring their stability. However, to reduce such variability, a standard microdilution protocol defines precise conditions such as the phase of the bacterial culture (logarithmic), amount of the substances, incubation temperature (37 °C), and time (overnight) [153,154,155]. In the case of AMPs, the evaluation conditions at the laboratory scale are different from those of the final application (i.e., physiological). In addition to that, analog to the emulsifiers in that the stabilization of emulsions depends on the oil phase substances, the effectiveness of the AMPs strongly depends on the type of microorganism used for the experiment. It is worth noting that even if an attractive MIC is measured for an AMP, this fails to assure that it effectively translates into the final clinical application. Something similar happens for emulsifiers in the case of the surface tension and the subsequent assays to measure emulsions formation where despite helping identifying possible candidates the evaluation in the complexity of real environments might lead to different performance results. The availability of standard properties allows us to select potential candidate molecules and to find and understand structure–performance relations. For example, from the analysis of AMP databases, it has been observed that the majority of AMPs have a positive net charge provided mainly by the presence of Lysine and Arginine residues, which was later related to their capacity to interact with the negatively charged membranes of bacteria [156]. From the screening of a large number of 12-residue peptides, the sequence LRLLR in positions 5–9 has been shown to provide the translocation ability, which is useful to transport molecules inside cells [120].

The approach discussed above for AMPs could be potentially applied for emulsifying peptides. The value of the surface tension at the CMC and 25 °C may be considered the standard property to compare the interfacial activity of peptides. This value has been already reported for peptides with different sizes and conformations, as shown in Table 1. Moreover, it has also been obtained from surface tension vs. concentration plots, as in the case of conventional surfactants. For the measurement of the emulsion stabilization, the turbiscan stability index (TSI) provided by the devices from the company Formulaction [157], or another turbidimetric property based on noninvasive techniques may be used as standard parameters. For example, TSI accounts for the change over time of the transmittance and absorbance in the emulsion that is caused by any destabilization phenomena, and the evaluation is completed employing a transparent vial in which the system is not perturbed such that the obtained values only reflect changes in the emulsion stability. Additionally, transmittance, absorbance, and backscattering plots along the height of the emulsion allow a comprehensive analysis of different destabilizing phenomena and their extents. Since the behavior of emulsifiers changes depending on the type of oil phase, TSI should be provided as a function of the used organic solvent, the same way as the MIC in AMPs is published specifically for a certain type of microorganism.

Once databases with standardized property values become available, new design approaches could be implemented for emulsifying peptides, as has been the case in other fields where such databases exist. Some examples of those strategies include the “ab initio” design to find common patterns step by step that respond to optimal functionalities of interest [156], the identification of new templates to find and study structure–performance relationships [158], and the improvement of a particular function by adding or replacing residues to a peptide or protein, based on machine learning models [159].

Additional to design techniques that rely on property databases, the other methods described here to select and design peptide emulsifiers have also produced important results. The use of protein hydrolysates to make emulsions and find the fractions of the protein with the best emulsifying behavior is a potent approach when the interest is to produce a mixture of peptides that have the ability to form and stabilize an emulsion. The advantage of this method is that the selection of the hydrolysate proceeds by evaluation in a real emulsion system; hence, the selected mixture has higher chances of properly working in the final application. However, this process is limited because it demands investment in reagents, equipment, time, personnel, and involves risks. Even when many studies have been conducted on the emulsification capacity of protein hydrolysates, hardly ever the peptides responsible for it are identified, and most times the studies just provide the frequency of the amino acids in the best hydrolysate fraction. This frequency information provides few insights into the mechanistic details because this parameter varies substantially by the presence of large peptides at the interface that might contribute insignificantly to the emulsifier activity, in other words, more potent but shorter peptides might be masked in this approach. Furthermore, possible synergistic effects emerging from the peptides contained in the hydrolysate can improve emulsification but might pass unattended by this analysis. In conclusion, more studies and suitable experimental techniques are needed to simplify the isolation and identification of the peptides with emulsifying behavior to gather more relevant information before a more rational approach to their design, selection, and production.

Computational tools tackle some of the disadvantages exposed in the identification and obtention of emulsifying peptides from proteins. Savings in time and material resources make these methods attractive for peptide design. In addition, the structure of the functional peptides and the structure–performance relations can be modeled in silico. However, implementing some of the computational tools for each step in the process is challenging and, depending on the ultimate purpose, might not fully replace experimental validation. For example, during the protein cleavage stage, some peptides detected in vitro after the enzyme treatment might be different from those predicted by computational cutter tools [85,122]. These differences exist because the databases of enzymes are only based on their specificity toward the primary sequence and fail to consider the secondary and tertiary structures, which have been reported to limit the cleavage process. Another reason for these differences is that most simulators fail to consider the effect of pH, temperature, time, and other types of interactions between molecules [122]. Finally, the use of MD simulations to predict the properties of peptides might be significantly hampered by the shortness of the time scales accessible, which limit the possibility of observing other phenomena like the formation of supramolecular structures. This causes that important discrepancies between experiments and simulations are observable for complex systems [65]. Coarse-grained models have emerged as an attempt to overcome the time-scale limitations caused by the important computational resources needed to simulate long-scale phenomena. Therefore, the growing computational power observed over the last decade worldwide has encouraged scientists to scale up the complexity of simulated systems with remarkable results.

Another technique discussed here is the de novo design. This method mainly relies on assembling long peptides with amphipathic secondary structures and predicting their emulsifier behavior; however, the identified structure–performance relationships are largely incomplete. For example, the phenomena concerning the interaction of some amino acids of the peptide chain with the interface still remain obscure, as well as the underlying patterns or amino acid sequences responsible for the reduction of the interfacial tension or the stabilization of the emulsions. This knowledge gap can be bridged by increasing the availability of property data and by designing novel experimental approaches for the rapid experimental screening of peptide libraries.

Finally, the route of design consisting of peptide crosslinking with facial amphipathic structure at the interface takes advantage of the possibility of altering the intermolecular attractive forces. However, the most well-characterized and studied method is the one put forward by Dexter et al., in which metallic cations were employed for the crosslinking of peptides at the interface of emulsion droplets [59]. The presence of these metallic cations narrows down the possible applications of the obtained emulsions since some industries (e.g., food, cosmetics, and pharma) might require metal-free products. For example, the commonly used Ni and Co ions can be toxic for humans depending on the concentration and oxidation state [160,161]. An interesting approach to avoiding high toxicity would be to exploit changes in salinity or pH to modify the intermolecular forces. However, even if salinity and pH variations can improve the stability of the emulsions, it is not certain that they can stabilize emulsions better than metallic cations crosslinking.

## 7. Conclusions

This review presented the principles underlying the formation and stabilization of emulsions. Here, we attempted to link these principles with the mechanistic understanding required to explain the properties responsible for conferring peptides the ability to lower the interfacial tension of oil–water systems and stabilize emulsions by steric and electrostatic forces. The comprehensive analysis, which primarily focused on the molecular structure of peptides, began with the simplest head–tail types of conventional emulsifiers, then it was gradually scaled to amino acids, and finally to peptides. We covered the most recent developments in the understanding of structure–performance correlations and the attempts to elucidate intricate details by experimental and in silico approaches. Furthermore, analogies with conventional emulsifiers were used to make more understandable explanations of what would be required to begin to understand the emulsifying behavior of peptides and rationally design it. Conditions of the system that affect this behavior, are discussed and the different methodologies to discover and design emulsifier peptides are also addressed. Even when different paths have been used to discover and design peptides with this function, each one of them faces challenges. Additionally, other routes that have been applied in other fields remain still unexplored for emulsifier peptides.

The discovery of emulsifying peptides from proteins has been widely used to discover and design emulsifying peptides. The experimental procedure requires more studies whose characterization techniques allow to draw conclusions about the peptides involved in the stabilization of the emulsions. This characterization is more adequately developed by the use of computational tools because the knowledge of possible emulsifying peptides is the starting point to address their production and testing in emulsions. However, the models used have drawbacks, and integration of simulation and experimental techniques seems to be the way that yields better results in finding new emulsifier peptides. The success of computational methods to identify good emulsifier peptides from proteins depends on the use of structure–performance relationships, whose understanding is vital to effectively predict the good emulsifying behavior of a peptide. These relations keen the computational tools and other methodologies to design emulsifier peptides such as the de novo design, whose capacity to design effective molecules is still very imprecise and require more analysis of the interaction of peptides with the interface in terms of the molecular structure, forces, amino acids, and patterns of amino acids or sequences. In fact, some authors increased the molecular forces by establishing facial amphipathicity in peptides and metal-binding capacity, which allowed them to crosslink with metallic cations and generate more cohesive oil–water interfaces. Even when this methodology allowed designing peptides with excellent formation and stabilization capacity, the application is limited by the toxicity of metallic cations, and other force-modifying alternatives deserve to be explored to stabilize emulsions with peptides.

This review also proposed the possibility to apply new emulsifier design methodologies based on property data management. These possibilities are justified in the results that these methodologies have yielded in other fields such as AMPs. For the development of this, emulsifier databases should be created with standard properties as a function of the molecular structure; to apply correlations or mathematical models that allow us to predict and design peptides, as well as to discover structure–performance relations that support the methodologies mentioned above. Concerning the creation of the databases, standard procedures were proposed to evaluate the interfacial activity of peptides and the capability to stabilize emulsions, such as the measurement of the γ_CMC_ at 25 °C and the TSI values, respectively. Finally, once the databases are created, the artificial intelligence techniques and mathematical models can be applied to discover new structure–performance relationships and optimize the emulsifier properties of interest. In the long run, we expect to produce a set of recommendations and a framework to enable emerging applications in the field of truly biodegradable product formulations. This is of the utmost importance due to stringent requirements increasing every day, imposed by regulatory bodies regarding the use of components of natural origin and low toxicity. Novel emulsifying peptides are exceedingly attractive candidates to fulfill these criteria. In line with these prospects, in the near future, we should be able to have new families of products in the cosmetic, food, home care, agrochemical, and pharma industries that do not include the use of surfactants or emulsifiers of petrochemical origin.

## References

[1] Hack B., Egger H., Uhlemann J., Henriet M., Wirth W., Vermeer A.W.P., Duff D. (2012). Advanced agrochemical formulations through encapsulation strategies?. Chem. Ing. Tech..

[2] Cussler E.L., Moggridge G.D. (2011). Chemical Product Design.

[3] Mattei M., Kontogeorgis G.M., Gani R. (2014). A comprehensive framework for surfactant selection and design for emulsion based chemical product design. Fluid Phase Equilib..

[4] Rosen M.J. (2004). Surfactants and Interfacial Phenomena.

[5] Khan B.A., Akhtar N., Khan H.M.S., Waseem K., Mahmood T., Rasul A., Iqbal M., Khan H. (2011). Basics of pharmaceutical emulsions: A review. Afr. J. Pharm. Pharmacol..

[6] Rehfeld S.J. (1967). The effects of initial surfactant concentration and emulsification time upon the particle size and distribution of benzene-in-water emulsions. J. Colloid Interface Sci..

[7] NG K.M., Gani R., Dam-Johansen K., NG K.M., Gani R., Dam-Johansen K. (2007). Chemical Product Design: Towards a Perspective through Case Studies. Chapter 1: Chemical Product Design—A Brief Overview.

[8] Usaid A. (2014). Emulsion and it’s Applications in Food Processing-A Review. Int. J. Eng. Res. Appl..

[9] Akbari S., Abdurahman N.H., Yunus R.M., Fayaz F., Alara O.R. (2018). Biosurfactants—a new frontier for social and environmental safety: A mini review. Biotechnol. Res. Innov..

[10] Rufino R.D., de Luna J.M., de Campos Takaki G.M., Sarubbo L.A. (2014). Characterization and properties of the biosurfactant produced by Candida lipolytica UCP 0988. Electron. J. Biotechnol..

[11] Tao W., Lin J., Wang W., Huang H., Li S. (2019). Designer bioemulsifiers based on combinations of different polysaccharides with the novel emulsifying esterase AXE from Bacillus subtilis CICC 20034. Microb. Cell Fact..

[12] Hayes D.G., Hayes D.G., Kitamoto D., Solaiman D., Ashby R. (2009). Biobased Surfactants Overview and Industrial State-of-the-Art. Biobased Surfactants and Detergents.

[13] Smith B.V., Ierapepritou M.G. (2010). Integrative chemical product design strategies: Reflecting industry trends and challenges. Comput. Chem. Eng..

[14] Cardona Jaramillo J.E.C., Achenie L.E., Álvarez O.A., Carrillo Bautista M.P., González Barrios A.F. (2020). The multiscale approach to the design of bio-based emulsions. Curr. Opin. Chem. Eng..

[15] Saito M., Ogasawara M., Chikuni K., Shimizu M. (1995). Synthesis of a Peptide Emulsifier with an Amphiphilic Structure. Biosci. Biotechnol. Biochem..

[16] McClements D.J., Bai L., Chung C. (2017). Recent Advances in the Utilization of Natural Emulsifiers to Form and Stabilize Emulsions. Annu. Rev. Food Sci. Technol..

[17] Uzoigwe C., Burgess J.G., Ennis C.J., Rahman P.K.S.M. (2015). Bioemulsifiers are not biosurfactants and require different screening approaches. Front. Microbiol..

[18] García-Moreno P.J., Gregersen S., Nedamani E.R., Olsen T.H., Marcatili P., Overgaard M.T., Andersen M.L., Hansen E.B., Jacobsen C. (2020). Identification of emulsifier potato peptides by bioinformatics: Application to omega-3 delivery emulsions and release from potato industry side streams. Sci. Rep..

[19] Poon S., Clarke A.E., Schultz C.J. (1999). Structure-function analysis of the emulsifying and interfacial properties of apomyoglobin and derived peptides. J. Colloid Interface Sci..

[20] De Faria A.F., Teodoro-Martinez D.S., De Oliveira Barbosa G.N., Gontijo Vaz B., Serrano Silva Í., Garcia J.S., Tótola M.R., Eberlin M.N., Grossman M., Alves O.L. (2011). Production and structural characterization of surfactin (C 14/Leu7) produced by Bacillus subtilis isolate LSFM-05 grown on raw glycerol from the biodiesel industry. Process Biochem..

[21] McClements D.J., Gumus C.E. (2016). Natural emulsifiers—Biosurfactants, phospholipids, biopolymers, and colloidal particles: Molecular and physicochemical basis of functional performance. Adv. Colloid Interface Sci..

[22] Dave N., Joshi T. (2017). A Concise Review on Surfactants and Its Significance. Int. J. Appl. Chem..

[23] Costa C., Medronho B., Filipe A., Mira I., Lindman B., Edlund H., Norgren M. (2019). Emulsion formation and stabilization by biomolecules: The leading role of cellulose. Polymers.

[24] Holmberg K., Jönsson B., Kronberg B., Lindman B. (2003). Surfactant and Polymers in Aqueous Solution.

[25] Farn R.J. (2007). Chemistry and Technology of Surfactants.

[26] Tadros T. (2013). Encyclopedia of Colloid and Interface Science.

[27] Kong X., Zhou H., Qian H. (2007). Enzymatic preparation and functional properties of wheat gluten hydrolysates. Food Chem..

[28] Malcolm A., Dexter A., Middelberg A. (2007). Peptide surfactants (Pepfactants) for switchable foams and emulsions. Asia-Pac. J. Chem. Eng..

[29] McClements D.J. (2007). Critical review of techniques and methodologies for characterization of emulsion stability. Crit. Rev. Food Sci. Nutr..

[30] Huibers P.D.T., Lobanov V.S., Katritzky A.R., Shah D.O., Karelson M. (1996). Prediction of critical micelle concentration using a quantitative structure-property relationship approach. 1. Nonionic surfactants. Langmuir.

[31] Márquez N., Bravo B., Ysambertt F., Chávez G., Subero N., Salager J.L. (2003). Analysis of polyethoxylated surfactants in microemulsion-oil-water systems: III. Fractionation and partitioning of polyethoxylated alcohol surfactants. Anal. Chim. Acta.

[32] Nakamura A., Muramatsu M. (1977). Complex formation between N-dodecyl-β-alanine and sodium alkylsulfate as reflected in coadsorption from mixed solution. J. Colloid Interface Sci..

[33] Belton J., Twidle H. (1940). The Surface Tensions of Amino-Acid Solutions. Trans. Faraday Soc..

[34] Rodríguez D.M., Romero C.M. (2017). Surface Tension of Glycine, Alanine, Aminobutyric Acid, Norvaline, and Norleucine in Water and in Aqueous Solutions of Strong Electrolytes at Temperatures from (293.15 to 313.15) K. J. Chem. Eng. Data.

[35] Raza M.A., Hallett P.D., Liu X., He M., Afzal W. (2019). Surface Tension of Aqueous Solutions of Small-Chain Amino and Organic Acids. J. Chem. Eng. Data.

[36] Yiase S.G. (2015). Amino acids as potential emulsifiers in stabilizing oil/water emulsions. Int. J. Innov. Sci. Res..

[37] Black C.D., Popovich N.G. (1981). A study of intravenous emulsion compatibility: Effects of dextrose, amino acids, and selected electrolytes. Drug Intell. Clin. Pharm..

[38] Imura T., Nakayama M., Taira T., Sakai H., Abe M., Kitamoto D. (2015). Interfacial and emulsifying properties of soybean peptides with different degrees of hydrolysis. J. Oleo Sci..

[39] Sewald N., Jakubke H.-D. (2002). Peptides: Chemistry and Biology.

[40] Fauchère J.-L., Pliska V. (1983). Hydrophobic parameters II of amino acid side-chains from the partitioning of N-acetyl-amino acid amides. Eur. J. Med. Chem..

[41] Li J., Wang J., Zhao Y., Zhou P., Carter J., Li Z., Waigh T.A., Lu J.R., Xu H. (2020). Surfactant-like peptides: From molecular design to controllable self-assembly with applications. Coord. Chem. Rev..

[42] Edwards-Gayle C.J.C., Hamley I.W. (2017). Self-assembly of bioactive peptides, peptide conjugates, and peptide mimetic materials. Org. Biomol. Chem..

[43] Zou Y., Tu B., Yu L., Zheng Y., Lin Y., Luo W., Yang Y., Fang Q., Wang C. (2019). Peptide conformation and oligomerization characteristics of surface-mediated assemblies revealed by molecular dynamics simulations and scanning tunneling microscopy. RSC Adv..

[44] Xue Y., He L., Middelberg A.P.J., Mark A.E., Poger D. (2014). Determining the structure of interfacial peptide films: Comparing neutron reflectometry and molecular dynamics simulations. Langmuir.

[45] Dexter A.F., Middelberg A.P.J. (2008). Peptides as functional surfactants. Ind. Eng. Chem. Res..

[46] James J., Mandal A.B. (2011). Micelle formation of Tyr-Phe dipeptide and Val-Tyr-Val tripeptide in aqueous solution and their influence on the aggregation of SDS and PEO-PPO-PEO copolymer micelles. Colloids Surf. B Biointerfaces.

[47] Scholberg H.M., Guenthner R.A., Coon R.I. (1953). Surface chemistry of fluorocarbons and their derivatives. J. Phys. Chem..

[48] Middelberg A.P.J., Dexter A.F. (2016). Peptide Networks. International Patent.

[49] Saiani A., Mohammed A., Frielinghaus H., Collins R., Hodson N., Kielty C.M., Sherratt M.J., Miller A.F. (2009). Self-assembly and gelation properties of α-helix versus β-sheet forming peptides. Soft Matter.

[50] Hamley I.W. (2011). Self-assembly of amphiphilic peptides. Soft Matter.

[51] Bai S., Pappas C., Debnath S., Frederix P.W.J.M., Leckie J., Fleming S., Ulijn R.V. (2014). Stable emulsions formed by self-assembly of interfacial networks of dipeptide derivatives. ACS Nano.

[52] Scott G.G., McKnight P.J., Tuttle T., Ulijn R.V. (2016). Tripeptide Emulsifiers. Adv. Mater..

[53] Girardet J.M., Debomy L., Courthaudon J.L., Miclo L., Humbert G., Gaillard J.L. (2000). Viscoelastic properties of oil-water interfaces covered by bovine β-casein tryptic peptides. J. Dairy Sci..

[54] Rahali V., Chobert J.M., Haertlé T., Guéguen J. (2000). Emulsification of chemical and enzymatic hydrolysates of β-lactoglobulin: Characterization of the peptides adsorbed at the interface. Nahr. Food.

[55] Lee S.W., Shimizu M., Kaminogawa S., Yamauchi K. (1987). Emulsifying properties of peptides obtained from the hydrolyzates of β-casein. Agric. Biol. Chem..

[56] Dickinson E. (1997). Properties of Emulsions Stabilized with Milk Proteins: Overview of Some Recent Developments. J. Dairy Sci..

[57] Davis J.P., Doucet D., Foegeding E.A. (2005). Foaming and interfacial properties of hydrolyzed β-lactoglobulin. J. Colloid Interface Sci..

[58] Kilara A., Panyam D. (2003). Peptides from Milk Proteins and Their Properties. Crit. Rev. Food Sci. Nutr..

[59] Dexter A.F., Middelberg A.P.J. (2007). Switchable peptide surfactants with designed metal binding capacity. J. Phys. Chem. C.

[60] Middelberg A.P.J., Radke C.J., Blanch H.W. (2000). Peptide interfacial adsorption is kinetically limited by the thermodynamic stability of self association. Proc. Natl. Acad. Sci. USA.

[61] Tan H.L., Curtis R. (2017). Inter-relation of surface tension and optical turbidity in self-assembled peptide amphiphiles. Biointerface Res. Appl. Chem..

[62] Hong Y., Lau L.S., Legge R.L., Chen P. (2004). Critical self-assembly concentration of an ionic-complementary peptide EAK16-I. J. Adhes..

[63] Persaud D.R., Dalgleish D.G., Nadeau L., Gauthier S. (2000). Isolation and purification of serum and interfacial peptides of a trypsinolyzed β-lactoglobulin oil-in-water emulsion. J. Chromatogr. B Biomed. Sci. Appl..

[64] Fernández-Niño M., Rojas L., Cifuentes J., Torres R., Ordonez A., Cruz J.C., Vargas E.F., Pradilla D., Solano O.Á., Barrios A.G. (2019). Insights into the behavior of six rationally designed peptides based on Escherichia coli’s OmpA at the water-dodecane interface. PLoS ONE.

[65] Aguilera-Segura S.M., Núñez Vélez V., Achenie L., Álvarez Solano O., Torres R., González Barrios A.F. (2016). Peptides design based on transmembrane Escherichia coli’s OmpA protein through molecular dynamics simulations in water–dodecane interfaces. J. Mol. Graph. Model..

[66] Shigeri Y., Yasuda A., Hagihara Y., Nishi K., Watanabe K., Imura T., Inagaki H., Haramoto Y., Ito Y., Asashima M. (2015). Identification of novel peptides from amphibian (Xenopus tropicalis) skin by direct tissue MALDI-MS analysis. FEBS J..

[67] Acquah C., Di Stefano E., Udenigwe C.C. (2018). Role of hydrophobicity in food peptide functionality and bioactivity. J. Food Bioact..

[68] Perez-Riverol Y., Audain E., Millan A., Ramos Y., Sanchez A., Vizcaíno J.A., Wang R., Müller M., Machado Y.J., Betancourt L.H. (2012). Isoelectric point optimization using peptide descriptors and support vector machines. J. Proteom..

[69] Turgeon S.L., Gauthier S.F., Paquin P. (1992). Emulsifying Property of Whey Peptide Fractions as a Function of pH and ionic Strength. J. Food Sci..

[70] Lam R.S.H., Nickerson M.T. (2013). Food proteins: A review on their emulsifying properties using a structure-function approach. Food Chem..

[71] Alashi A.M., Blanchard C.L., Mailer R.J., Agboola S.O., Mawson J.A., Aluko R.E. (2018). Influence of enzymatic hydrolysis, pH and storage temperature on the emulsifying properties of canola protein isolate and hydrolysates. Int. J. Food Sci. Technol..

[72] Lee S.J., Choi S.J., Li Y., Decker E.A., McClements D.J. (2011). Protein-stabilized nanoemulsions and emulsions: Comparison of physicochemical stability, lipid oxidation, and lipase digestibility. J. Agric. Food Chem..

[73] Keowmaneechai E., McClements D.J. (2002). Effect of CaCl2 and KCl on physiochemical properties of model nutritional beverages based on whey protein stabilized oil-in-water emulsions. J. Food Sci..

[74] Adnoju R. (2014). Whey protein peptides as dual-functional ingredients in food nanoemulsions. Ph.D. Thesis.

[75] Martínez-Maqueda D., Miralles B., Cruz-Huerta E., Recio I. (2013). Casein hydrolysate and derived peptides stimulate mucin secretion and gene expression in human intestinal cells. Int. Dairy J..

[76] Claustre J., Toumi F., Trompette A., Jourdan G., Guignard H., Chayvialle J.A., Plaisancié P. (2002). Effects of peptides derived from dietary proteins on mucus secretion in rat jejunum. Am. J. Physiol. Gastrointest. Liver Physiol..

[77] Cudennec B., Ravallec-Plé R., Courois E., Fouchereau-Peron M. (2008). Peptides from fish and crustacean by-products hydrolysates stimulate cholecystokinin release in STC-1 cells. Food Chem..

[78] El Hatmi H., Jrad Z., Khorchani T., Jardin J., Poirson C., Perrin C., Cakir-Kiefer C., Girardet J.M. (2016). Identification of bioactive peptides derived from caseins, glycosylation-dependent cell adhesion molecule-1 (GlyCAM-1), and peptidoglycan recognition protein-1 (PGRP-1) in fermented camel milk. Int. Dairy J..

[79] Balti R., Bougatef A., Sila A., Guillochon D., Dhulster P., Nedjar-Arroume N. (2015). Nine novel angiotensin I-converting enzyme (ACE) inhibitory peptides from cuttlefish (Sepia officinalis) muscle protein hydrolysates and antihypertensive effect of the potent active peptide in spontaneously hypertensive rats. Food Chem..

[80] Lafarga T., Aluko R.E., Rai D.K., O’Connor P., Hayes M. (2016). Identification of bioactive peptides from a papain hydrolysate of bovine serum albumin and assessment of an antihypertensive effect in spontaneously hypertensive rats. Food Res. Int..

[81] Lin P., Wong J.H., Ng T.B. (2010). A defensin with highly potent antipathogenic activities from the seeds of purple pole bean. Biosci. Rep..

[82] Liu Z., Dong S., Xu J., Zeng M., Song H., Zhao Y. (2008). Production of cysteine-rich antimicrobial peptide by digestion of oyster (Crassostrea gigas) with alcalase and bromelin. Food Control.

[83] Popineau Y., Huchet B., Larré C., Bérot S. (2002). Foaming and emulsifying properties of fractions of gluten peptides obtained by limited enzymatic hydrolysis and ultrafiltration. J. Cereal Sci..

[84] Moro A., Báez G.D., Ballerini G.A., Busti P.A., Delorenzi N.J. (2013). Emulsifying and foaming properties of β-lactoglobulin modified by heat treatment. Food Res. Int..

[85] Bechaux J., Gatellier P., Le Page J.F., Drillet Y., Sante-Lhoutellier V. (2019). A comprehensive review of bioactive peptides obtained from animal byproducts and their applications. Food Funct..

[86] Drozłowska E., Weronis M., Bartkowiak A. (2020). The influence of thermal hydrolysis process on emulsifying properties of potato protein isolate. J. Food Sci. Technol..

[87] Benítez R., Ibarz A., Pagan J. (2008). Hidrolizados de proteína: Procesos y aplicaciones. Acta Bioquim. Clin. Latinoam..

[88] Chew L.Y., Toh G.T., Ismail A., Kuddus M. (2018). Application of proteases for the production of bioactive peptides. Enzymes in Food Biotechnology: Production, Applications, and Future Prospects.

[89] Jeewanthi R.K.C., Lee N.K., Paik H.D. (2015). Improved functional characteristics of whey protein hydrolysates in food industry. Korean J. Food Sci. Anim. Resour..

[90] Padial-Domínguez M., Espejo-Carpio F.J., Pérez-Gálvez R., Guadix A., Guadix E.M. (2020). Optimization of the emulsifying properties of food protein hydrolysates for the production of fish oil-in-water emulsions. Foods.

[91] Huang X.L., Catignani G.L., Swaisgood H.E. (1996). Improved Emulsifying Properties of β-Barrel Domain Peptides Obtained by Membrane-Fractionation of a Limited Tryptic Hydrolysate of β-Lactoglobulin. J. Agric. Food Chem..

[92] Turgeon S.L., Sanchez C., Gauthier S.F., Paquin P. (1996). Stability and rheological properties of salad dressing containing peptidic fractions of whey proteins. Int. Dairy J..

[93] Park B.Y., Yoon K.Y. (2019). Functional properties of enzymatic hydrolysate and peptide fractions from perilla seed meal protein. Pol. J. Food Nutr. Sci..

[94] Chabanon G., Chevalot I., Framboisier X., Chenu S., Marc I. (2007). Hydrolysis of rapeseed protein isolates: Kinetics, characterization and functional properties of hydrolysates. Process Biochem..

[95] Schmidt M.M., da Fontoura A.M., Vidal A.R., Dornelles R.C.P., Kubota E.H., de Mello R.O., Cansian R.L., Demiate I.M., de Oliveira C.S. (2020). Characterization of hydrolysates of collagen from mechanically separated chicken meat residue. Food Sci. Technol..

[96] Ling Z., Ai M., Zhou Q., Guo S., Zhou L., Fan H., Cao Y., Jiang A. (2020). Fabrication egg white gel hydrolysates-stabilized oil-in-water emulsion and characterization of its stability and digestibility. Food Hydrocoll..

[97] Padial-Domínguez M., Espejo-Carpio F.J., García-Moreno P.J., Jacobsen C., Guadix E.M. (2020). Protein derived emulsifiers with antioxidant activity for stabilization of omega-3 emulsions. Food Chem..

[98] Dai L., Hinrichs J., Weiss J. (2020). Emulsifying properties of acid-hydrolyzed insoluble protein fraction from Chlorella protothecoides: Formation and storage stability of emulsions. Food Hydrocoll..

[99] Van der Ven C., Gruppen H., De Bont D.B.A., Voragen A.G.J. (2001). Emulsion properties of casein and whey protein hydrolysates and the relation with other hydrolysate characteristics. J. Agric. Food Chem..

[100] Caessens P.W.J.R., Visser S., Gruppen H., Voragen A.G.J. (1999). β-Lactoglobulin hydrolysis. 1. Peptide composition and functional properties of hydrolysates obtained by the action of plasmin, trypsin, and Staphylococcus aureus V8 protease. J. Agric. Food Chem..

[101] Schröder A., Berton-Carabin C., Venema P., Cornacchia L. (2017). Interfacial properties of whey protein and whey protein hydrolysates and their influence on O/W emulsion stability. Food Hydrocoll..

[102] Gao Y., Li J., Chang C., Wang C., Yang Y., Su Y. (2019). Effect of enzymatic hydrolysis on heat stability and emulsifying properties of egg yolk. Food Hydrocoll..

[103] Bao Z.J., Zhao Y., Wang X.Y., Chi Y.J. (2017). Effects of degree of hydrolysis (DH) on the functional properties of egg yolk hydrolysate with alcalase. J. Food Sci. Technol..

[104] Liu J., Lyu F., Zhou X., Wang B., Wang X., Ding Y. (2015). Preparation of Skipjack Tuna (Katsuwonus pelamis) Protein Hydrolysate Using Combined Controlled Enzymatic Hydrolysis and Glycation for Improved Solubility and Emulsifying Properties. J. Food Nutr. Res..

[105] Quan T.H., Benjakul S. (2019). Production and characterisation of duck albumen hydrolysate using enzymatic process. Int. J. Food Sci. Technol..

[106] Wu W.U., Hettiarachchy N.S., Qi M. (1998). Hydrophobicity, solubility, and emulsifying properties of soy protein peptides prepared by papain modification and ultrafiltration. JAOCS J. Am. Oil Chem. Soc..

[107] Kim H.J., Choi S.J., Shin W.S., Moon T.W. (2003). Emulsifying properties of bovine serum albumin-galactomannan conjugates. J. Agric. Food Chem..

[108] Saito M., Yin L.J., Kobayashi I., Nakajima M. (2006). Comparison of stability of bovine serum albumin-stabilized emulsions prepared by microchannel emulsification and homogenization. Food Hydrocoll..

[109] Kiosseoglou V., Perdikis A. (1994). Stability of bovine serum albumin-stabilized olive oil-in-water emulsions and the role of the oil minor surface-active lipids. Food Hydrocoll..

[110] Saito M., Chikuni K., Monma M., Shimizu M. (1993). Emulsifying and Oil-binding Properties of Bovine Serum Albumin and Its Enzymatic Hydrolyzate. Biosci. Biotechnol. Biochem..

[111] Cheema M., Hristov A.N., Harte F.M. (2017). The binding of orally dosed hydrophobic active pharmaceutical ingredients to casein micelles in milk. J. Dairy Sci..

[112] Lorient D., Closs B., Courthaudon J.L. (1989). Surface properties of the bovine casein components: Relationships between structure and foaming properties. J. Dairy Res..

[113] Courthaudon J.L., Girardet J.M., Campagne S., Rouhier L.M., Campagna S., Linden G., Lorient D. (1999). Surface active and emulsifying properties of casein micelles compared to those of sodium caseinate. Int. Dairy J..

[114] Shimizu M., Lee S.W., Kaminogawa S., Yamauchi K. (1984). Emulsifying Properties of an N-Terminal Peptide Obtained from the Peptic Hydrolyzate of αs1-Casein. J. Food Sci..

[115] Zhai J., Miles A.J., Pattenden L.K., Lee T.H., Augustin M.A., Wallace B.A., Aguilar M.I., Wooster T.J. (2010). Changes in β-lactoglobulin conformation at the oil/water interface of emulsions studied by synchrotron radiation circular dichroism spectroscopy. Biomacromolecules.

[116] Qian C., Decker E.A., Xiao H., McClements D.J. (2011). Comparison of biopolymer emulsifier performance in formation and stabilization of orange oil-in-water emulsions. JAOCS J. Am. Oil Chem. Soc..

[117] Singh H., Sarkar A. (2011). Behaviour of protein-stabilised emulsions under various physiological conditions. Adv. Colloid Interface Sci..

[118] Tu M., Cheng S., Lu W., Du M. (2018). Advancement and prospects of bioinformatics analysis for studying bioactive peptides from food-derived protein: Sequence, structure, and functions. TrAC Trends Anal. Chem..

[119] Rathinakumar R., Wimley W.C. (2010). High-throughput discovery of broad-spectrum peptide antibiotics. FASEB J..

[120] Fuselier T., Wimley W.C. (2017). Spontaneous Membrane Translocating Peptides: The Role of Leucine-Arginine Consensus Motifs. Biophys. J..

[121] Selvaraj C., Sakkiah S., Tong W., Hong H. (2018). Molecular dynamics simulations and applications in computational toxicology and nanotoxicology. Food Chem. Toxicol..

[122] Iwaniak A., Darewicz M., Mogut D., Minkiewicz P. (2019). Elucidation of the role of in silico methodologies in approaches to studying bioactive peptides derived from foods. J. Funct. Foods.

[123] University of Warmia and Mazury in Olsztyn BIOPEP-UWM. http://www.uwm.edu.pl/biochemia/index.php/en/biopep.

[124] Swiss Institute of Bioinformatics PeptideCutter. https://web.expasy.org/peptide_cutter/.

[125] Agyei D., Tsopmo A., Udenigwe C.C. (2018). Bioinformatics and peptidomics approaches to the discovery and analysis of food-derived bioactive peptides. Anal. Bioanal. Chem..

[126] Bioware. http://bioware.ucd.ie/~compass/biowareweb/.

[127] Jahangiri R., Soltani S., Barzegar A. (2014). A Review of QSAR Studies to Predict Activity of ACE Peptide Inhibitors. Pharm. Sci..

[128] Veerasamy R., Rajak H., Jain A., Sivadasan S., Varghese C.P., Agrawal R.K. (2011). Validation of QSAR Models—Strategies and Importance. Int. J. Drug Des. Disocovery.

[129] Ulmschneider J.P., Ulmschneider M.B. (2018). Molecular Dynamics Simulations Are Redefining Our View of Peptides Interacting with Biological Membranes. Acc. Chem. Res..

[130] Álvarez Vanegas M., Macías Lozano A., Núñez Vélez V., Garcés Ferreira N., Castro Barrera H., Álvarez Solano O., González Barrios A.F. (2013). Molecular dynamics approach to investigate the coupling of the hydrophilic-lipophilic balance with the configuration distribution function in biosurfactant-based emulsions. J. Mol. Model..

[131] She A.Q., Gang H.Z., Mu B.Z. (2012). Temperature influence on the structure and interfacial properties of surfactin micelle: A molecular dynamics simulation study. J. Phys. Chem. B.

[132] Copps J., Murphy R.F., Lovas S. (2008). Molecular dynamics simulations of peptides. Methods Mol. Biol..

[133] Wei T., Huang T., Qiao B., Zhang M., Ma H., Zhang L. (2014). Structures, dynamics, and water permeation free energy across bilayers of lipid a and its analog studied with molecular dynamics simulation. J. Phys. Chem. B.

[134] Chen C.H., Starr C.G., Troendle E., Wiedman G., Wimley W.C., Ulmschneider J.P., Ulmschneider M.B. (2019). Simulation-Guided Rational de Novo Design of a Small Pore-Forming Antimicrobial Peptide. J. Am. Chem. Soc..

[135] Farrotti A., Bocchinfuso G., Palleschi A., Rosato N., Salnikov E.S., Voievoda N., Bechinger B., Stella L. (2015). Molecular dynamics methods to predict peptide locations in membranes: LAH4 as a stringent test case. Biochim. Biophys. Acta Biomembr..

[136] Copps J., Murphy R.F., Lovas S., Otvos L. (2008). Molecular Dynamics Simulations of Peptides. Peptide-Based Drug Design. Methods in Molecular Biology.

[137] Rossi G., Fuchs P.F.J., Barnoud J., Monticelli L. (2012). A coarse-grained MARTINI model of polyethylene glycol and of polyoxyethylene alkyl ether surfactants. J. Phys. Chem. B.

[138] Liu X., Li Y., Tian S., Yan H. (2019). Molecular Dynamics Simulation of Emulsification/Demulsification with a Gas Switchable Surfactant. J. Phys. Chem. C.

[139] Miyamoto H., Rein D.M., Ueda K., Yamane C., Cohen Y. (2017). Molecular dynamics simulation of cellulose-coated oil-in-water emulsions. Cellulose.

[140] Aguilera-Segura S.M., Macias A., Carrero D., Vargas W., Vives-Florez M., Castro H., Alvarez O., Gonzalez A., Castillo F.L., Cristancho M., Isaza G., Pinzón A., Rodríguez C.J.M. (2014). Escherichia coli´s OmpA as Biosurfactant for Cosmetic Industry: Stability Analysis and Experimental Validation Based on Molecular Simulations. Advances in Computational Biology, Proceedings of the 2nd Colombian Congress on Computational Biology and Bioinformatics(CCBCOL).

[141] Alting A.C., Pouvreau L., Giuseppin M.L.F., van Nieuwenhuijzen N.H., Williams P.A., Phillips G.O. (2011). Potato proteins. Handbook of Food Proteins.

[142] Schmidt J.M., Damgaard H., Greve-Poulsen M., Larsen L.B., Hammershøj M. (2018). Foam and emulsion properties of potato protein isolate and purified fractions. Food Hydrocoll..

[143] Hanson J.A., Chang C.B., Graves S.M., Li Z., Mason T.G., Deming T.J. (2008). Nanoscale double emulsions stabilized by single-component block copolypeptides. Nature.

[144] Wimley W.C. (2010). Describing the Mechanism of Antimicrobial Peptide Action with the Interfacial Activity Model. ACS Chem. Biol..

[145] Castelletto V., Edwards-Gayle C.J.C., Hamley I.W., Barrett G., Seitsonen J., Ruokolainen J. (2019). Peptide-Stabilized Emulsions and Gels from an Arginine-Rich Surfactant-like Peptide with Antimicrobial Activity. ACS Appl. Mater. Interfaces.

[146] Cantor S., Vargas L., Rojas O.E.A., Yarce C.J., Salamanca C.H., Oñate-Garzón J. (2019). Evaluation of the antimicrobial activity of cationic peptides loaded in surface-modified nanoliposomes against foodborne bacteria. Int. J. Mol. Sci..

[147] Fairman R., Chao H.-G., Mueller L., Lavoie T.B., Shen L., Novotny J., Matsueda G.R. (1995). Characterization of a new four-chain coiled-coil: Influence of chain length on stability. Protein Sci..

[148] Fukunishi Y., Tateishi T., Suzuki M. (1996). Octane/water interfacial tension calculation by molecular dynamics simulation. J. Colloid Interface Sci..

[149] Jones D.B., Middelberg A.P.J. (2002). Mechanical properties of interfacially adsorbed peptide networks. Langmuir.

[150] Dexter A.F., Malcolm A.S., Middelberg A.P.J. (2006). Reversible active switching of the mechanical properties of a peptide film at a fluid-fluid interface. Nat. Mater..

[151] Malcolm A.S., Dexter A.F., Katakdhond J.A., Karakashev S.I., Nguyen A.V., Middelberg A.P.J. (2009). Tuneable control of interfacial rheology and emulsion coalescence. ChemPhysChem.

[152] Dexter A.F. (2010). Interfacial and emulsifying properties of designed β-strand peptides. Langmuir.

[153] Lab H. Centre for Microbial Diseases and Immunity Research (CMDR). http://cmdr.ubc.ca/bobh/methods/methodsall.html.

[154] Yu K., Park K., Kim Y., Kang S.W., Shin S.Y., Hahm K.S. (2002). Solution structure of a cathelicidin-derived antimicrobial peptide, CRAMP as determined by NMR spectroscopy. J. Pept. Res..

[155] Wang G., Li Y., Li X. (2005). Correlation of three-dimensional structures with the antibacterial activity of a group of peptides designed based on a nontoxic bacterial membrane anchor. J. Biol. Chem..

[156] Mishra B., Wang G. (2012). Ab Initio Design of Potent Anti-MRSA Peptides based on Database Filtering Technology. J. Chem. Soc..

[157] Formulaction Turbiscan Stability Index. https://www.formulaction.com/en/knowledge-center/turbiscan-stability-index.

[158] Menousek J., Mishra B., Hanke M.L., Heim C.E., Kielian T., Wang G. (2012). Database screening and in vivo efficacy of antimicrobial peptides against methicillin-resistant Staphylococcus aureus USA300. Int. J. Antimicrob. Agents.

[159] Han X., Ning W., Ma X., Wang X., Zhou K. (2020). Improving protein solubility and activity by introducing small peptide tags designed with machine learning models. Metab. Eng. Commun..

[160] Genchi G., Carocci A., Lauria G., Sinicropi M.S., Catalano A. (2020). Nickel: Human health and environmental toxicology. Int. J. Environ. Res. Public Health.

[161] Czarnek K., Terpilowska S., Siwicki A.K. (2015). Selected aspects of the action of cobalt ions in the human body. Cent. Eur. J. Immunol..

